# The chemistry of cationic polyphosphorus cages – syntheses, structure and reactivity

**DOI:** 10.1039/c4cs00019f

**Published:** 2014-04-17

**Authors:** Michael H. Holthausen, Jan J. Weigand

**Affiliations:** a Department of Chemistry , University of Toronto , Toronto , Canada . Email: m.holthausen@utoronto.ca; b Fachrichtung Chemie und Lebensmittelchemie , TU Dresden , Professur für Koordinationschemie , Dresden , Germany . Email: jan.weigand@tu-dresden.de ; Tel: +49 (351) 468-42800

## Abstract

The aim of this review is to provide a comprehensive view of the chemistry of cationic polyphosphorus cages. The synthetic protocols established for their preparation, which are all based on the functionalization of P_4_, and their intriguing follow-up chemistry are highlighted. In addition, this review intends to foster the interest of the inorganic, organic, catalytic and material oriented chemical communities in the versatile field of polyphosphorus cage compounds. In the long term, this is envisioned to contribute to the development of new synthetic procedures for the functionalization of P_4_ and its transformation into (organo-)phosphorus compounds and materials of added value.

## Abstract 2

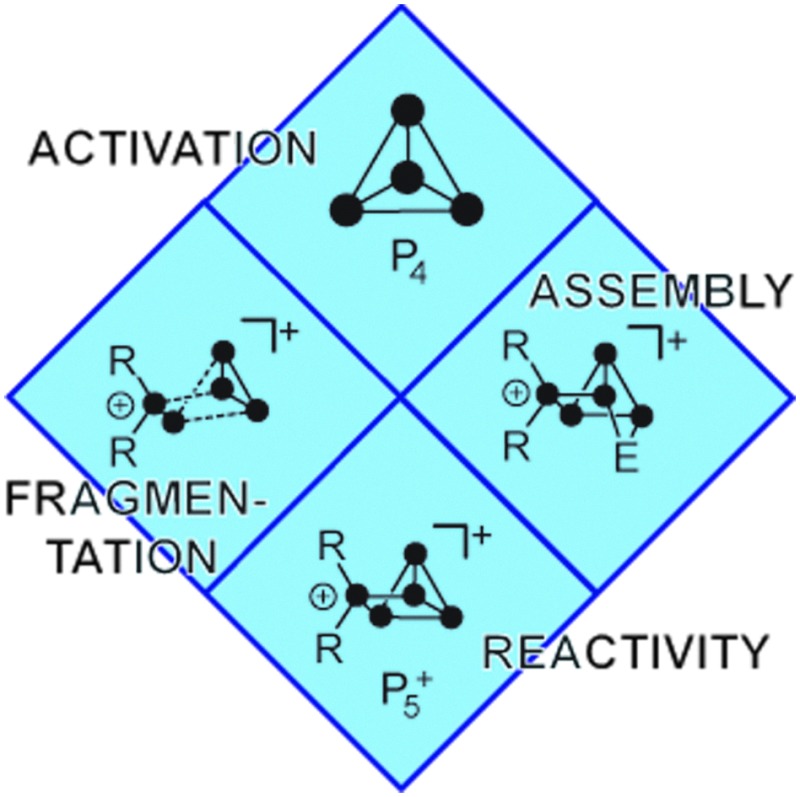
The aim of this review is to provide a comprehensive view of the chemistry of cationic polyphosphorus cages.

## 1. Introduction

Discovering novel pathways for the activation and transformation of white phosphorus (P_4_) is important for the ongoing search for new, systematic entries to polyphosphorus and organo-phosphorus compounds. Especially in the realm of polyphosphorus cations methods for the preparation of species featuring a high P to substituent ratio are rare. In contrast, a systematic access to highly substituted cations R_*n*_P_*m*_ (*n* > *m*) is achieved with synthetic protocols mainly based on the utilization of neutral *catena* or *cyclic* polyphosphanes R_*n*_P_*m*_.^1^ Protocols for phosphorus-rich cations R_*n*_P_*m*_ (*n* < *m*) often involve P_1_-precursors and are based on the reduction of either P–Cl^2^ or P–H bonds.^3^ Multiple P–P bonds are formed in these reactions giving access to elaborate P–P bonded frameworks. However, in most cases the reaction outcome is unpredictable which hampers the targeted preparation of polyphosphorus cations. Thus, a synthetic approach that takes advantage of the tetrahedral P_4_ framework should allow for a targeted and systematic assembly of phosphorus-rich cations R_*n*_P_*m*_ (*n* < *m*). Additionally, the application of P_4_ in such conversions is of high interest, since it constitutes an important raw material in industrial chemistry and is produced on a megaton-scale nowadays.^4^ The desire to develop synthetic protocols for the more sustainable production of P-containing bulk chemicals has sparked significant academic and industrial research efforts within the last decades. Progress in the areas of transition metal^5^ and main group^6^ mediated P_4_ activation has been reviewed several times. However, no account was given so far on the importance of P_4_ as a starting material for the preparation of polyphosphorus cations.

The aim of this review is to provide a comprehensive view of the chemistry of cationic polyphosphorus cages.

The synthetic protocols established for their preparation, which are all based on the functionalization of P_4_, and their intriguing follow-up chemistry are highlighted. In addition, this review intends to foster the interest of the inorganic, organic, catalytic and material oriented chemical communities in the versatile field of polyphosphorus cage compounds. In the long term, this is envisioned to contribute to the development of new synthetic procedures for the functionalization of P_4_ and its transformation into (organo-)phosphorus compounds and materials of added value.

In the following, black dots denote P atoms in order to provide easily comprehensible drawings of complex polyphosphorus frameworks for the reader. These frameworks may give rise to complicated, sometimes higher order, spin systems in their ^31^P NMR spectra. Their designation is derived by assigning letters in alphabetical order starting with the resonance at the highest field. The spin systems were considered to be higher order and consecutive letters are assigned if Δ*δ*(P_i_P_ii_)/^*n*^ *J*(P_i_P_ii_) < 10. For Δ*δ*(P_i_P_ii_)/^*n*^ *J*(P_i_P_ii_) > 10, the spin system is considered to be pseudo first order and the assigned letters are separated. However, if a group of similar compounds is discussed, only one spin system is mentioned for the sake of clarity. All cationic polyphosphorus cages presented here are obtained by functionalization of P_4_. Mostly, phosphenium ions or cationic phosphorus species which formally serve as a phosphenium ion source are used for this functionalization. It is of high importance for the reader to be aware of the general reactivity pattern of P_4_ and the general characteristics of phosphenium ions. Thus, a brief insight into both fields is given in the first two sections.

## 2. P_4_ activation pathways

**Fig. 1:**
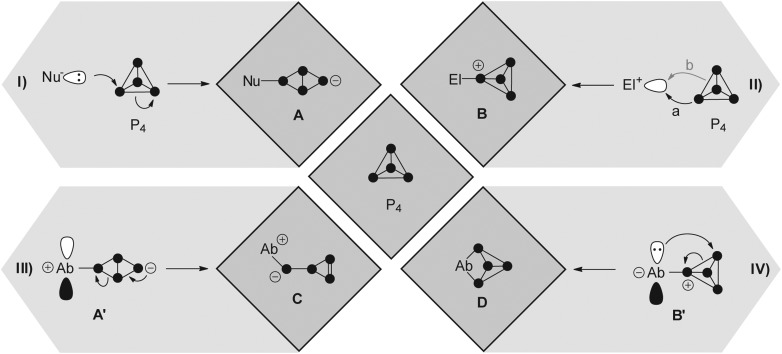
Generalized reactions of P_4_ with nucleophiles (I, Nu^–^), electrophiles (II, El^+^), predominantly nucleophilic ambiphiles (III, Ab), and predominantly electrophilic ambiphiles (IV, Ab); **A–D** illustrate structural motifs obtained after reaction with the aforementioned species.

In order to gain an in depth understanding of the reactions of P_4_ and main group element compounds, it is crucial to understand the properties of the P_4_ tetrahedron. The bonding in P_4_ is almost “cluster-like”, strongly delocalized and mostly effected through 3p atomic orbitals. Interestingly, P_4_ shows spherical aromaticity and is virtually unstrained despite acute bond angles of 60°.^7^ Generalized reactions of P_4_ with nucleophiles (Nu^–^), electrophiles (El^+^) and ambiphiles (Ab) are shown in Fig. 1. Radical reactions involving P_4_ are excluded. A nucleophile (Nu^–^) interacts with the LUMO of P_4_ (–1.8 eV),^7^ which leads to the rupture of a P–P bond giving butterfly-type bicyclo[1.1.0]tetraphosphane **A** (Fig. 1I). The reactions of P_4_ with nucleophiles were intensely investigated using an array of organo-alkali and organo-alkali earth reagents.^6^ However, in many cases the formation of a derivative of **A** only constitutes the first step of a reaction sequence which ultimately leads to the degradation of P_4_ to P_1_-compounds.^6^ Only a few reactions involving a selective cleavage of only a single bond in the P_4_ tetrahedron are reported.

**Fig. 2:**
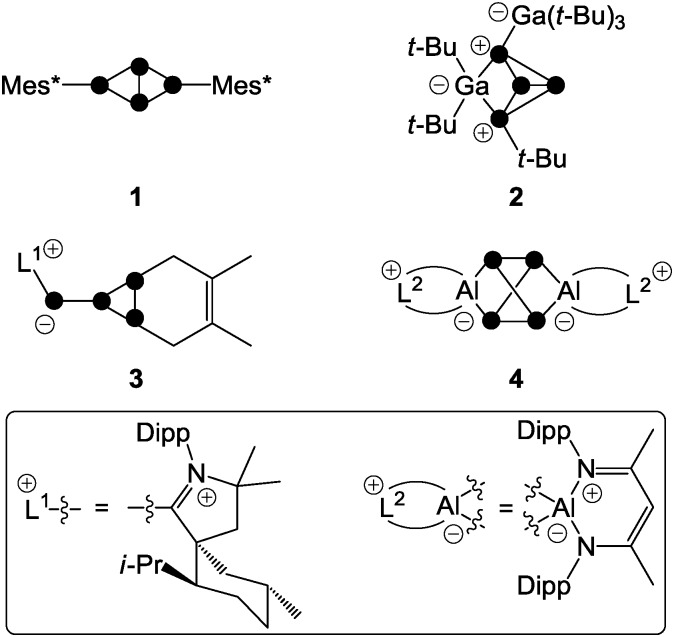
Examples of polyphosphorus compounds obtained by the functionalization of P_4_ by nucleophiles (**1**), electrophiles (**2**), predominantly nucleophilic ambiphiles (**3**), and predominantly electrophilic ambiphiles (**4**).

One is the reaction of Mes*Li (Mes* = 2,4,6-tri-*tert*-butylphenyl) with one equivalent of P_4_ yielding a tetraphosphanide intermediate of type **A**. Subsequent reaction with Mes*Br yields the butterfly-type species **1** (Fig. 2).^8^ Further degradation of **1** is prevented by the sterically demanding Mes*-groups. Nucleophiles based on silicon, main group 5 or main group 6 elements were also employed.^6^ An electrophile may attack at a non-bonding orbital of lone pair character (HOMO – 6, –7.5 eV)^7^ which results in the formation of compounds of type **B** (Fig. 1II a). Alternatively, an electrophile may attack at a bonding orbital at one of the edges of the tetrahedron (HOMO, –6.7 eV; Fig. 1II b). However, this mode of attack is commonly less productive for main group element centered electrophiles and is not depicted. In total, only very few reactions with electrophiles were reported due to the low nucleophilicity of P_4_.^9^ One example constitutes the reaction of P_4_ with two equivalents of the sterically encumbered Lewis acid Ga(*t*-Bu)_3_. This yields compound **2**; however, mechanistic details regarding its formation were not reported (Fig. 2).^10^

The utilization of ambiphilic main group element compounds (Ab) for the activation of P_4_ represents a rather new synthetic approach. Reactions of P_4_ with ambiphiles can be divided into two categories assuming an asynchronous process with two consecutive steps. The first category comprises reactions of P_4_ with predominantly nucleophilic ambiphiles. Similar to the reactions of P_4_ and nucleophiles, intermediate **A′** is obtained in the first step of the reaction. Subsequently, **A′** rearranges to *cyclo*-triphosphirene derivative **C** (Fig. 1III). The rearrangement is attributed to the propensity of Ab to accept electron density from the adjacent P atom which formally leads to the formation of an Ab–P double bond. Carbenes are ambiphiles with a predominantly nucleophilic character.^11^ Two types of carbenes, *i.e.* N-heterocyclic carbenes (NHC) and cyclic or acyclic alkyl amino carbenes (*c*AAC or *a*AAC), were investigated in reactions with P_4_ by the research group of Bertrand.^12^ The formation of an intermediate of type **A′** was confirmed by DFT calculations^12^ and of type **C** by trapping experiments with 2,3-dimethylbutadiene yielding [2+4] *cyclo*-addition product **3** (Fig. 2, *e.g.* L^1^ = cAAC).

The second category comprises reactions of P_4_ with predominantly electrophilic ambiphiles. By analogy with the reactions involving electrophiles, the first step of the reaction is an electrophilic attack of Ab yielding an intermediate **B′** (Fig. 1). Subsequently, **B′** rearranges to a bicyclo[1.1.0]tetraphosphane **D** featuring a bridging Ab moiety (IV). This reaction sequence equals the formal insertion of the ambiphile in one of the P–P bonds of the P_4_ tetrahedron. P_4_ functionalization involving a predominantly electrophilic ambiphile is an experimentally more widespread approach. Monovalent group 13 element compounds with the oxidation state +I are a class of substances that are widely used in such transformations.^13^ The first type of such a structural motif was achieved by Roesky and coworkers by reacting P_4_ with two equivalents of Al(I) compound AlL^2^ (L^2^ = CH{(CMe)(2,6-i-Pr_2_C_6_H_3_N)}_2_).^13^ The formal insertion of AlL^2^ into one P–P bond of P_4_ is assumed to give an intermediate of type **B′** in the first step. However, the insertion of a second equivalent of AlL^2^ into the opposing P–P bond of the P_4_ tetrahedron occurs rapidly yielding the two-fold insertion product **4** (Fig. 2). In addition, P_4_ activation by predominantly electrophilic silylenes,^14^ disilenes,^15^ phosphasilenes,^16^ and a bis(stannylene)^17^ was reported. Reactions of P_4_ with phosphenium cations (R_2_P^+^) are also classified as P_4_ functionalization with predominantly electrophilic ambiphiles. They will be thoroughly discussed within this review from an experimental as well as a mechanistic point of view.

## 3. Syntheses and characteristics of phosphenium ions

**Fig. 3:**
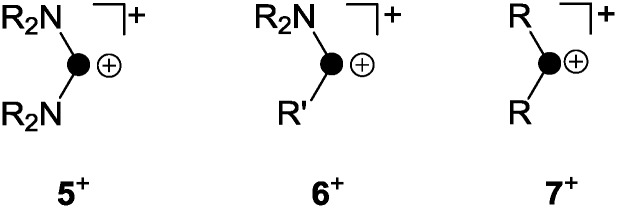
Distinct types of phosphenium ions featuring two (**5** ^+^) or one (**6** ^+^) stabilizing amino-substituents and elusive, non-stabilized phosphenium ion **7** ^+^ (R, R′ = alkyl, aryl).

The term phosphenium ion describes a cation featuring a di-coordinated, positively polarized P atom.^18^ Phosphenium ions reveal a lone pair of electrons and a formally vacant p-type orbital, and thus, they constitute carbene analogues.^11^ The stability of phosphenium ions strongly depends on their substituents. While aryl- or alkylphosphenium ions R_2_P^+^ (**7** ^+^, Fig. 3) are strongly electrophilic and generally elusive, a large series of phosphenium ions bearing amino-substituents (R_2_N)_2_P^+^ (R = alkyl, aryl) are known.^18^ Three methods for their preparation are mainly reported throughout the literature. Halide abstraction from the corresponding halo-phosphane precursor is the most commonly used synthetic protocol.^18^ Further methods constitute the protolysis of P–N single bonds by Brønsted acids and the coordination of strong Lewis acids to P–N double bonds.^18^

The increased stability of phosphenium ions (R_2_N)_2_P^+^ (**5** ^+^, Fig. 3) stems from a lowered electrophilicity due to donation of π-electron density from the lone pair of electrons at the nitrogen atoms to the vacant p-type orbital at the P atom.^19^ Phosphenium ions of type **6** ^+^ featuring one amino-substituent are borderline cases between both of the aforementioned types and are only scarcely investigated. Only a few fully characterized derivatives are reported to date bearing either (pseudo-) halogens^20^ or sterically demanding aryl-moieties^21^ as the second substituent R′ on phosphorus (Fig. 3).

**Scheme 1:**
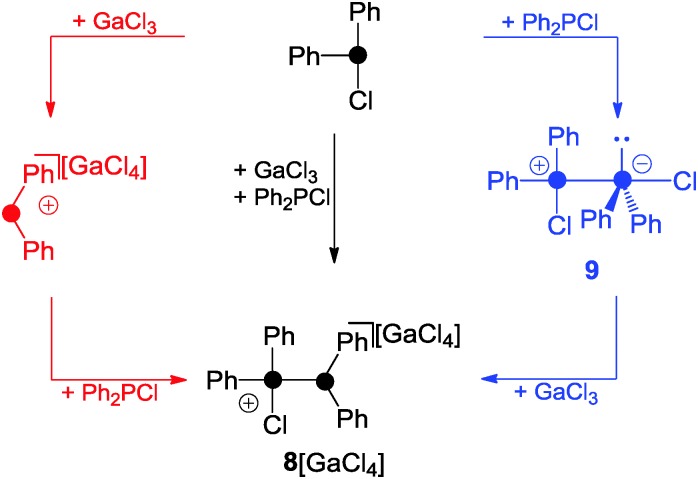
Synthesis of **8**[GaCl_4_] by the reaction of Ph_2_PCl and GaCl_3_ in 2 : 1 stoichiometry and possible reaction sequences giving **8**[GaCl_4_] either *via* free Ph_2_P^+^-phosphenium ion (red) or zwitterion **9** (blue).

A phosphenium ion bearing only alkyl- or aryl-substituents has not been isolated to date.^18^ The reaction of phosphanes bearing organo- and chloro-groups R_*n*_PCl_(3–*n*)_ (*n* = 1, 2) and a halide abstracting agent (*e.g.* Me_3_SiOTf, GaCl_3_, or AlCl_3_) in the appropriate stoichiometry usually results in the formation of phosphanylphosphonium ions.^1^ This is best exemplified by the reaction of Ph_2_PCl and GaCl_3_ in a 2 : 1 stoichiometry which yields **8**[GaCl_4_] (Scheme 1).^22^

**Scheme 2:**
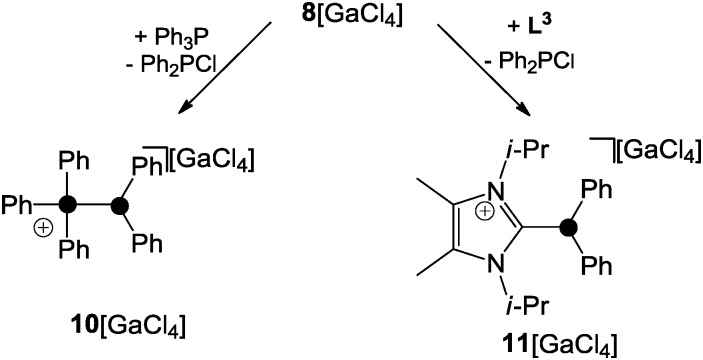
Substitution of the phosphoniumyl-moiety of **8** ^+^ by Lewis-bases.

Two mechanisms for the formation of **8** ^+^ are conceivable. Firstly, Ph_2_PCl reacts with GaCl_3_ as a halide abstracting agent giving a transient Ph_2_P^+^-phosphenium ion. This reacts with the second equivalent of Ph_2_PCl yielding **8** ^+^. The second and in the author's opinion more likely mechanism proceeds *via* the zwitterionic intermediate **9** which features a Ph_2_PCl molecule donating electron density from its lone pair of electrons to the lobes of the antibonding σ*(P–Cl) orbital of a second molecule of Ph_2_PCl. Subsequently, chloride abstraction by GaCl_3_ yields **8** ^+^ without an intermediary formation of a free Ph_2_P^+^-phosphenium ion. The phosphoniumyl-moiety in **8** ^+^ is easily substituted when **8** ^+^ is reacted with phosphanes of higher basicity than the leaving group.^1^ This is illustrated by the reaction of **8** ^+^ with Ph_3_P yielding **10** ^+^ and Ph_2_PCl (Scheme 2, left).^23^ Other Lewis bases are also suitable as nucleophiles. This is illustrated by the reaction of **8** ^+^ with 1,3-di-iso-propyl-4,5-dimethylimidazol-2-ylidene (L^3^) which yields the imidazoliumyl-substituted phosphane **11** ^+^.^23^

**Scheme 3:**
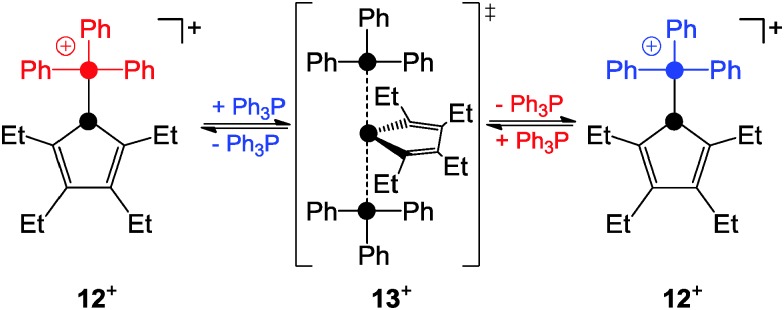
S_N_2-type substitution of the phosphoniumyl-moiety in **12** ^+^.

Detailed investigations of mixtures of phosphanyl-phosphonium ion **12** ^+^ and Ph_3_P revealed second-order kinetics for the exchange process of Ph_3_P consistent with a S_N_2-type pathway (Scheme 3).^24^

**Scheme 4:**
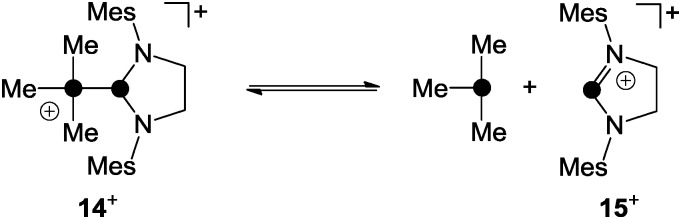
S_N_1-type dissociation of phosphanylphosphonium ion **14** ^+^.

This was further supported by quantum chemical calculations which suggested the phosphoranide-type transition state **13** ^+^ for the substitution process.^24^ In contrast, the phosphanyl-phosphonium ion **14** ^+^, which is formed *via* the reaction of phosphenium ion **15** ^+^ and PMe_3_, was reported to favour a dissociative S_N_1-type reaction pathway in substitution reactions (Scheme 4).^25^

For phosphanylphosphonium ions such as those described above the term “ligand stabilized phosphenium ions” is frequently used in the literature while the described substitution reactions are also called “ligand exchange” reactions.^1^ Independent of any such controversy, however, these distinct points of view are based on the labile P–P bond observed in phosphanylphosphonium ions. This allows for the transfer of R_2_P^+^-moieties (formally phosphenium ions) between distinct Lewis bases (*e.g.* phosphanes, carbenes or P_4_). Thus, for reasons of simplification, phosphanylphosphonium ions will be regarded as “sources of phosphenium ions”^1^ throughout this review.

**Fig. 4:**
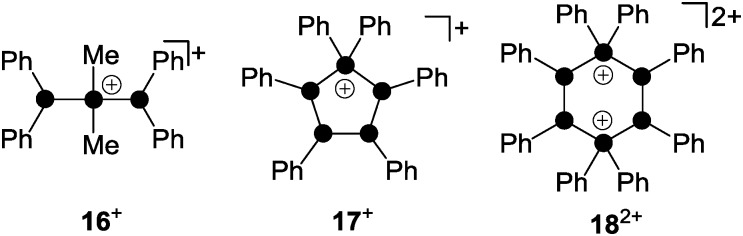
Polyphosphorus cations **16** ^+^, **17** ^+^ and **18** ^2+^ obtained *via* the formal insertion of R_2_P^+^-phosphenium ions (R = Me, Ph) into the P–P bond of (Ph_2_P)_2_, (PhP)_4_ and (PhP)_5_.

Phosphanylphosphonium ions were frequently used as phosphenium ion sources. The reaction of a mixture of Me_2_PCl and Me_3_SiOTf with diphosphane (Ph_2_P)_2_ gave diphosphanylphosphonium ion **16** ^+^ as a triflate salt (Fig. 4).^26^ Species **16** ^+^ is formally derived from the insertion of a Me_2_P^+^-phosphenium ion into the P–P bond of the diphosphane (Ph_2_P)_2_. Mixtures of Ph_2_PCl and Me_3_SiOTf with the *cyclo*-phosphanes (PhP)_4_ or (PhP)_5_ give in both cases the *cyclo*-tetraphosphanylphosphonium ion **17** ^+^.

A ring expansion is observed in the reaction with (PhP)_4_ whereas a 5-membered ring is retained in the reaction involving (PhP)_5_ *via* an unknown redistribution process.^26^ Both reactions proceed *via* the formal transfer of a Ph_2_P^+^-phosphenium ion from the intermediary formed phosphanylphosphonium ion **8** ^+^. In both cases **17** ^+^ is exclusively formed which demonstrates the thermodynamic preference of the five-membered ring over the six-membered alternative. The highly reactive, cyclic six-membered dication **18** ^2+^ is only obtained by employing a melt approach.^27^ Solvent-free mixtures of Ph_2_PCl and GaCl_3_ provide room temperature molten media. These melts represent a powerful source of phosphenium ions Ph_2_P^+^.^28^

## 4. Cationic homoleptic polyphosphorus cages

**Fig. 5:**
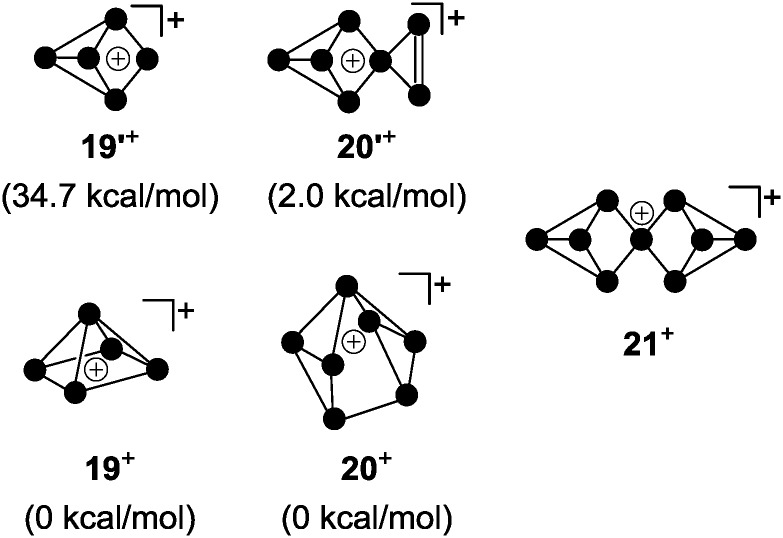
Anticipated structures of homoleptic, diamagnetic polyphosphorus cations **19** ^+^, **20** ^+^ and **21** ^+^.

For decades the investigation of homoleptic polyphosphorus cations was limited to mass spectroscopy^29^ and quantum chemical calculation^30^ in the gas phase. Homoleptic P_*n*_^+^ cations are paramagnetic if the number of P atoms *n* is even. In the case of an odd number of P atoms the respective cation is diamagnetic. In general, the paramagnetic series of polyphosphorus cations is less stable. In the odd-membered series, the smaller P_*n*_^+^ cations **19** ^+^ (*n* = 5) and **20** ^+^ (*n* = 7) may be described as electron-deficient Wade clusters whereas larger P_*n*_^+^-cages (*n* ≥ 9) feature electron-precise Zintl-type structures. According to Wade's rules, a square pyramidal structure is anticipated for cation **19** ^+^ (Fig. 5, *nido*-cluster). Such a structure was confirmed as the most stable isomer by means of quantum chemical calculations.^30*a*^ The structural motif of the second most stable isomer **19′** ^+^ (34.7 kcal mol^–1^ higher in energy) does not follow Wade's rules and shows a di-coordinated P atom. The most stable isomer of P_7_^+^-cage **20** ^+^ is a tricapped trigonal prism that is missing two of the capping vertices (*arachno*-cluster). A second isomer, which is only slightly higher in energy (**20′** ^+^, 2.0 kcal mol^–1^), shows the P_5_-cage motif of **19′** ^+^ and a three-membered P ring which are both fused by a bridging phosphonium moiety. The P_9_^+^-cage **21** ^+^, which is composed of two P_4_-moieties fused by a phosphonium moiety, is one of the most stable homoleptic polyphosphorus cations according to quantum chemical calculations (Fig. 5).^30*b*^

**Scheme 5:**
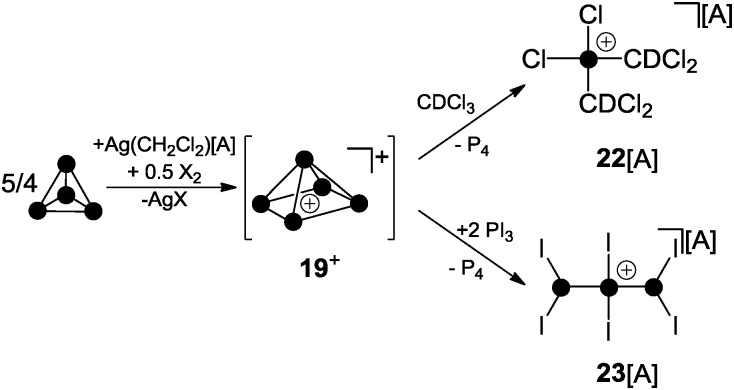
Oxidation of P_4_ with I_2_ or Br_2_ in the presence of Ag(CH_2_Cl_2_)[A] *via* P_5_^+^-cage intermediate **19** ^+^; A = Al(OC(CF_3_)_3_)_4_.

**Scheme 6:**
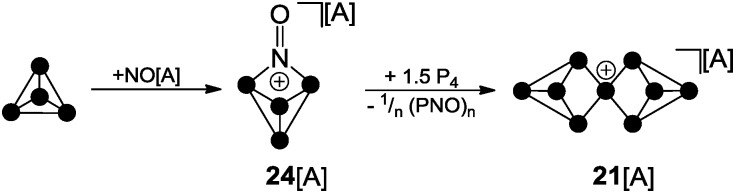
Reaction of P_4_ and NO[A] yielding P_4_NO^+^-cage **24**[A] and subsequent reaction with P_4_ yielding P_9_^+^-cage compound **21**[A]; A = Al(OC(CF_3_)_3_)_4_.

Krossing and co-workers were the first to report evidence for the existence of homoleptic polyphosphorus cations in the condensed phase.^31^ The attempted oxidation of P_4_ with I_2_ or Br_2_ in the presence of Ag(CH_2_Cl_2_)[A] (A = Al(OC(CF_3_)_3_)_4_) was suggested to proceed *via* the intermediary formation of P_5_^+^-cage cation **19** ^+^ (Scheme 5).^32^ However, cation **19** ^+^ is highly reactive and reacts with the solvent to give phosphonium ion **22** ^+^ as one of the main products. Cation **22** ^+^ forms *via* elimination of P_4_ and two-fold insertion into C–Cl bonds of CDCl_3_ molecules which was used as solvent. In the case of I_2_ as oxidant, P_4_ reacts partially to give PI_3_ which was suggested to react with intermediate **19** ^+^ to give P_4_ and the bis(phosphanyl)-substituted phosphonium ion **23** ^+^. Experimental evidence confirming the presence of **19** ^+^ in the reaction mixtures was not obtained; however, the suggested reaction pathways are in accordance with quantum chemical calculations.^32^ The nitrosonium salt [NO][A] (A = Al(OC(CF_3_)_3_)_4_) was also investigated as a possible one electron oxidant. However, the reaction of P_4_ with [NO][A] yields P_4_NO^+^-cage compound **24**[A] *via* insertion of the nitrosonium cation into a P–P bond (Scheme 6).^33^

Although X-ray structure determination of compound **24**[A] was not successful, the molecular structure is confirmed by spectroscopic data and computational investigations. The theoretical investigations suggested a two-step mechanism indicating the HOMO of P_4_ and a π*-type LUMO at NO^+^ as the interacting frontier orbitals (II b, Fig. 1).^33^ Similar results were obtained utilizing the carborate salt NO[HCB_11_Cl_11_].^34^ The reaction of P_4_NO^+^-cage compound **24**[A] with additional 1.5 equivalents of P_4_ was reported to yield P_9_^+^-cage compound **21**[A] which is the first isolated salt of a homoleptic phosphorus cation (Scheme 6).^35^

The reaction proceeds very likely *via* extrusion of 1/*n* (PNO)_*n*_ and intermediary formation of a P_3_^+^-species. The P_9_^+^ cation **21** ^+^ is obtained upon reaction of the latter with 1.5 equivalents of P_4_ *via* an unknown reaction mechanism. The ^31^P NMR spectrum of cation **21** ^+^ shows a characteristic A_2_A′_2_BC_2_C′_2_ spin system which confirms the *D*_2d_ symmetric Zintl-type structure. Despite the electron precise Lewis formula of eight neutral, three-coordinated and one cationic, four-coordinated P atom the charge is almost evenly distributed over all nine atoms according to quantum chemical calculations.^35^

## 5. Cationic polyphosphorus cages featuring halogen-substituents

**Scheme 7:**
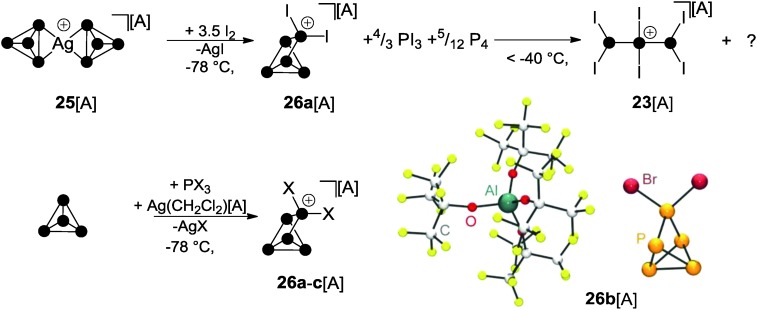
Oxidation of Ag(i) complex **25**[A] with I_2_ at low temperatures giving intermediary P_5_I_2_^+^-cage **26a** ^+^ (top), targeted syntheses of P_5_X_2_^+^-cages **26a–c** ^+^ (X = I, Br, Cl) by the reaction of P_4_, PX_3_ and Ag(CH_2_Cl_2_)[A] and molecular structure of **26b**[A] (bottom); A = Al(OC(CF_3_)_3_)_4_.

The oxidation of Ag(i) complex **25**[A] (A = Al(OC(CF_3_)_3_)_4_) featuring two intact P_4_ ligands with elemental iodine at low temperatures gives rise to interesting binary PI cations. The P_5_I_2_^+^-cage **26a** ^+^ was observed in the reaction mixture at –78 °C together with PI_3_ and P_4_ (Scheme 7).^36^ However, on raising the temperature above –40 °C, decomposition of **26a** ^+^ was observed, leading to the formation of P_3_I_6_^+^ (**23** ^+^) and unidentified by-products. A proposed reaction mechanism indicates the partial oxidation of the P_4_ ligands in **25** ^+^ by I_2_ to give PI_3_.^36^ The latter reacts with Ag[A] (A = Al(OC(CF_3_)_3_)_4_) *via* halide abstraction to give AgI and formally the phosphenium ion PI_2_^+^. This highly reactive, predominantly electrophilic ambiphile reacts with white phosphorus *via* insertion in one of the P–P bonds of the P_4_ tetrahedron yielding the P_5_I_2_^+^-cage **26a** ^+^. Likewise, according to the observations described in Section 3, a mechanism involving the formation of phosphanylphosphonium ion P_2_I_5_^+^ can also be considered. Here, P_2_I_5_^+^ is assumed to transfer a PI_2_^+^ phosphenium ion to P_4_ and, thus, serves as a phosphenium ion source. Upon warming the reaction mixture, the excess of PI_3_ reacts with P_4_ to yield diphosphane P_2_I_4_ in a conproportionation reaction. The diphosphane reacts with **26a** ^+^ *via* transfer of the phosphenium ion PI_2_^+^. This gives P_4_ and the P_3_I_6_^+^ cation **23** ^+^ which is formed upon insertion of the PI_2_^+^ ion into the P–P bond of P_2_I_4_. On the basis of these observations, a synthetic protocol for the targeted preparation of P_5_X_2_^+^-cages was developed (Scheme 7).^36,37^ Thus, white phosphorus reacts with PX_3_ (X = I, Br) in the presence of Ag(CH_2_Cl_2_)[A] as a halide abstracting agent and salts of cage cations **26a** ^+^ and **26b** ^+^ can be isolated in good yield. However, utilizing PCl_3_, the formation of the respective cation **26c** ^+^ was observed only in trace amounts since it readily decomposes in the reaction mixture.^38^ The molecular structure of **26b** ^+^ is shown in Scheme 7. The structural motif of the P_5_-core of the P_5_X_2_^+^-cage was unprecedented and was not previously observed as part of the many known polyphosphides and organo-polyphosphanes.

## 6. Cationic polyphosphorus cages featuring alkyl- and aryl-groups

**Fig. 6:**
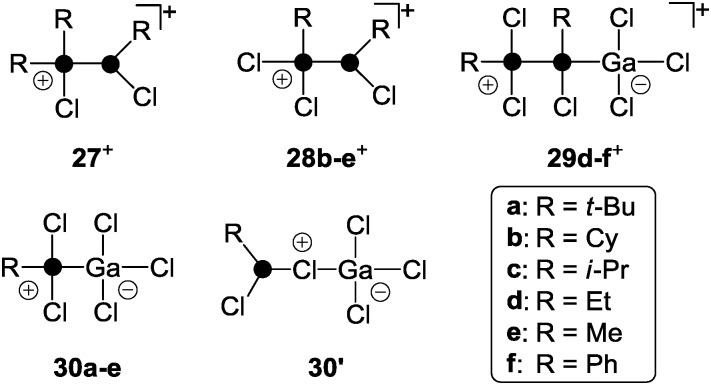
Phosphanylphosphonium ion derivatives **27** ^+^–**29** ^+^ and Lewis acid–base adduct **30** (classical) and **30′** (non-classical); R = alkyl, aryl.

A versatile approach to cationic polyphosphorus cages featuring alkyl- and aryl-groups represents the utilization of dichlorophosphanes RPCl_2_ (R = alkyl, aryl) instead of PX_3_ (X = I, Br, Cl).^39^ Mixtures of dichlorophosphanes RPCl_2_ and a strong Lewis acid (GaCl_3_, AlCl_3_) as a halide abstracting reagent can be utilized as the source for the phosphenium ion RPCl^+^. In the presence of P_4_, insertion into one of the P–P bonds takes place, giving access to a series of RP_5_Cl^+^-cages featuring distinct substituents R.^39^ Mixtures of dichlorophosphanes and AlCl_3_ were previously utilized for the *in situ* formation of phosphenium ion salts [RPCl][AlCl_4_] and subsequent syntheses of various phosphorus heterocycles.^40^ However, neither free phosphenium ions nor respective phosphenium ion sources could be verified. In some cases, the formation of Lewis acid–base complexes of the type *m*RPCl_2_·*n*AlCl_3_ (*n* = 1, 2; *m* = 1, 2) was suggested.^41^ Detailed investigations of mixtures of mono- and dichlorophosphanes in the presence of Lewis acids revealed the formation of chlorophosphanylchlorophosphonium ions of type **27** ^+^ (Fig. 6).^42^ In most cases, characteristic ^1^ *J*(PP) coupling constants were observed by ^31^P NMR spectroscopy at ambient temperature.

However, the spectra of mixtures of dichlorophosphanes and Lewis acids in CH_2_Cl_2_ were less informative and showed in most cases only broad resonances.^42^ A systematic study based on Raman and ^31^P NMR spectroscopy of mixtures of RPCl_2_ and GaCl_3_ in fluorobenzene applying varying stoichiometries gave important insight into these reactions.^39^ Depending on the ratio of the reactants and the substituent R in RPCl_2_, mixtures of the structurally distinct species **28** ^+^, **29** ^+^ and **30** were formed (Fig. 6).

The classical Lewis acid–base adducts of type **30** are only formed in reaction mixtures involving dichlorophosphanes RPCl_2_ featuring alkyl-substituents R. The formation of non-classical adducts of type **30′** is not observed and is unlikely according to quantum chemical calculations.^39^ This is further supported by the isolation and structural characterization of **30a** (R = *t*-Bu), which was proven to form a classical Lewis acid–base adduct. An increasing amount of phosphanylphosphonium ions of type **28** ^+^ is formed with decreasing steric demand of the substituent R (*t*-Bu > Cy > i-Pr). The formation of cations of type **29** ^+^ is observed when the basicity and the steric requirements of the dichlorophosphanes are further reduced (R = Et, Me, Ph). Such cations are the result of adduct formation between GaCl_3_ and the phosphane moiety of phosphanylphosphonium ions of type **28** ^+^. Most mixtures show dynamic exchange indicating a possible interconversion of species **28** ^+^, **29** ^+^ and **30**.^39^ The exchange rates of these processes strongly depends on the concentration of GaCl_3_. In the reaction mixtures equilibrium dissociation of the GaCl_4_^–^ anion to free GaCl_3_ and Cl^–^ occurs. The dynamic exchange is linked to these chloride anions which nucleophilically attack phosphanylphosphonium species yielding the phosphane starting materials in a back reaction. By using an excess of GaCl_3_ the GaCl_4_^–^ forms higher gallates (Ga_2_Cl_7_^–^ or Ga_3_Cl_10_^–^) and the concentration of free chloride anions is reduced.^43^

**Fig. 7:**
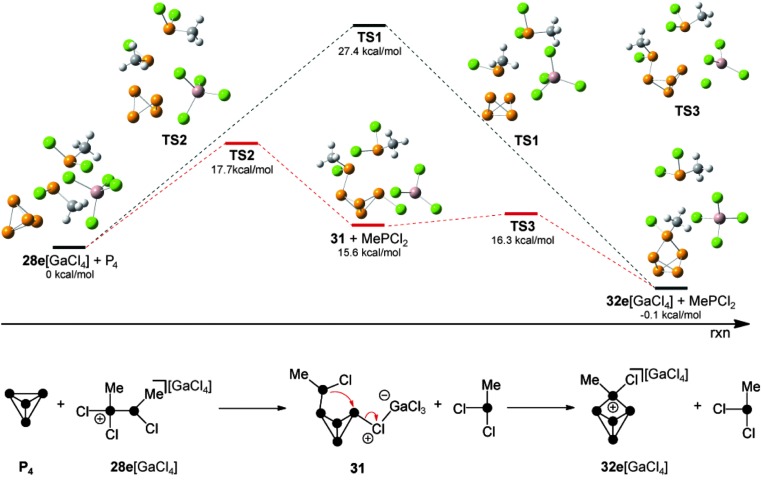
Calculated reaction pathways for the reaction of **28e**[GaCl_4_] and P_4_; calculated differences of the enthalpies at 298.15 K (Δ*H*_298_) are given for the optimized structures of MP2/6-31G(d) and the optimized structures of **28e**[GaCl_4_] + P_4_ were defined as 0 kcal mol^–1^.

Quantum chemical calculations were carried out to determine which of the observed species serves as the phosphenium ion source in a reaction with P_4_. According to these results,^44^ the formation of RP_5_Cl^+^-cages *via* a free phosphenium ion RPCl^+^ can be excluded. Attempts to calculate a feasible reaction mechanism from adducts **30** or **30′** as sources of phosphenium ions were not successful. Thus, the reaction of P_4_ with methyl-substituted phosphanylphosphonium derivative **28e** ^+^ was investigated (Fig. 7). A single step insertion of the phosphenium moiety into a P–P bond of the P_4_ tetrahedron is viable and the calculated energy profile of the reaction path is denoted in black. In addition, a two-step reaction pathway is feasible as well (energy profile is shown in red).

The two step reaction pathway proceeds *via* butterfly-type compound **31** as an intermediate (bottom, Fig. 7). The single step transfer of the phosphenium moiety in **28e**[GaCl_4_] and insertion thereof into a P–P bond of P_4_ shows an energy barrier of 27.4 kcal mol^–1^ (TS1) and is energetically viable. In the light of recent mechanistic studies on the reaction of isoelectronic silylenes with P_4_,^7*a*^ this is best understood as a combined electrophilic and nucleophilic attack of the phosphenium moiety. On the one hand the P–P bond of P_4_ (HOMO) nucleophilically attacks the p-type orbital of the phosphenium moiety. On the other hand the lone pair of electrons of the phosphenium moiety donates electron density to the LUMO of the P_4_ tetrahedron which corresponds to p-orbitals situated perpendicular to the P_4_ lone pairs.^7^ It was found that a lower barrier reaction pathway is possible if **28e** ^+^ does not act as a nucleophile. Instead, a chloro-substituent of the GaCl_4_^–^ anion nucleophilically attacks the P_4_ tetrahedron along with the electrophilic attack of the phosphenium moiety of **28e** ^+^ on P_4_. This leads to the slightly endothermic formation of the intermediate **31** (15.6 kcal mol^–1^) *via* transition state TS2 (17.7 kcal mol^–1^). Compound **31** reveals a butterfly-type structure featuring a chloro-substituent in an *exo*-position and a phosphanyl-substituent in an *endo*-position. Finally, **31** reacts *via* TS3 (16.3 kcal mol^–1^), which shows only a very low energy barrier. This step proceeds *via* the intramolecular nucleophilic attack of the phosphanyl-substituent on the chloro-substituted P atom. This eliminates the GaCl_4_^–^ anion and leads to the formation of the P_5_^+^-cage cation **32e** ^+^.

**Scheme 8:**
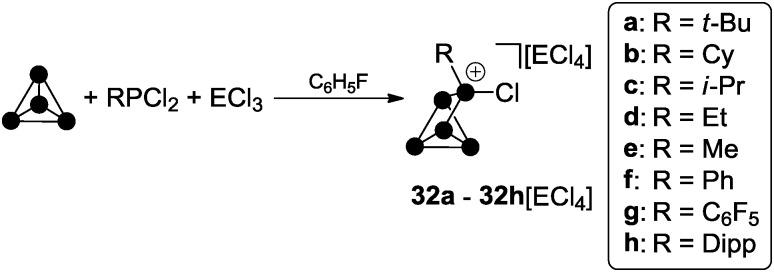
Preparation of compounds **32a–h**[ECl_4_] from P_4_, RPCl_2_ and ECl_3_ (E = Ga, Al; R = alkyl, aryl) in fluorobenzene.

Despite their different compositions 1 : 1 mixtures of RPCl_2_ and a Lewis acid ECl_3_ (E = Al, Ga) in fluorobenzene are potent sources of reactive phosphenium ion RPCl^+^ equivalents, which insert formally into P–P bonds of P_4_.^39^ Dissolution of P_4_ in these mixtures yielded white to yellowish precipitates of the corresponding RP_5_Cl^+^-cage salts for a large range of different alkyl- and aryl-substituents R (**32a–h**[GaCl_4_], Scheme 8).

**Fig. 8:**
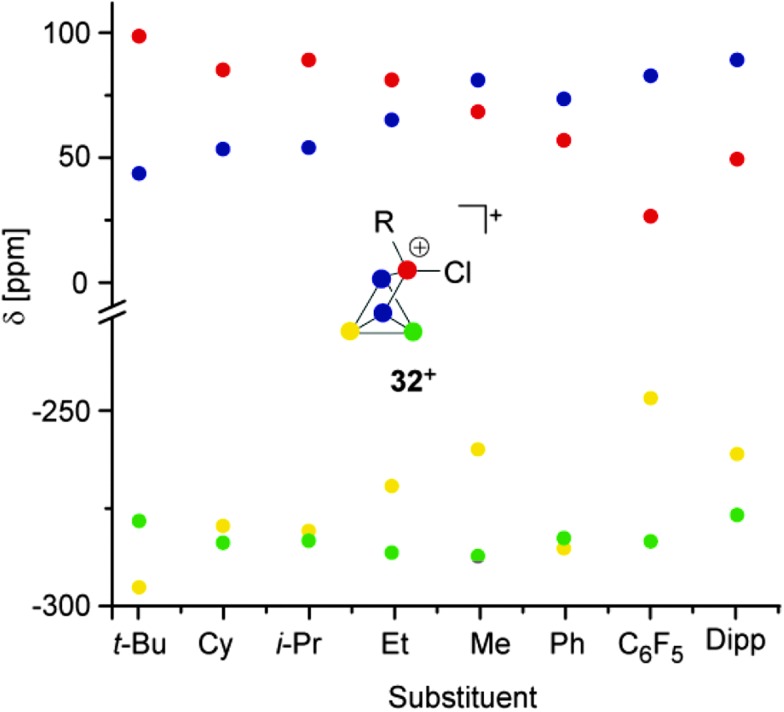
Plot of the ^31^P NMR chemical shifts of RP_5_Cl^+^-cages **32a-h** ^+^ *versus* their alkyl- or aryl-substituent.

**Fig. 9:**
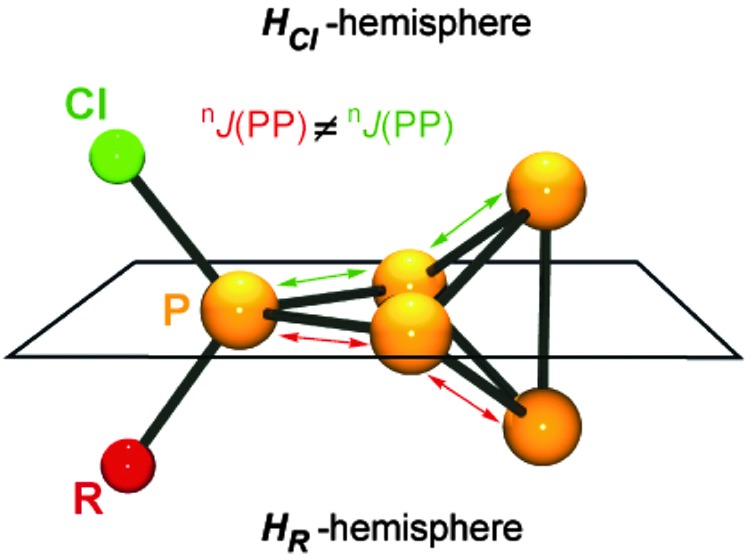
Definition of the H_Cl_- and H_R_-hemisphere in cations **32a–h** ^+^. The tetra-coordinated and the adjacent P atoms span a plane. The tri-coordinated P atom above the plane is within the H_Cl_-hemisphere, and the P atom below the plane is in the H_R_-hemisphere.

All compounds are obtained in almost quantitative yield and high purity. In contrast to the halogen-substituted species **26a–c**[A], they are stable in the solid state or when dissolved in non-coordinating solvents at ambient temperature.^36,37^ The cations **32a–h** ^+^ show characteristic ^31^P NMR spectra. Iterative line shape analysis of the observed spin systems gave chemical shifts and coupling constants in accordance with *C*_S_ symmetric RP_5_Cl^+^-cages with four chemically non-equivalent phosphorus nuclei. All cages possess a mirror plane which includes the tetra-coordinated P atom and both P atoms opposing the former. Due to the reduced symmetry compared to the *C*_2V_-symmetric P_5_X_2_^+^-cages **26a–c** ^+^ an ABM_2_X spin system is observed for **32a–d** ^+^ and an ABMX_2_ spin system for **32e–h** ^+^. Due to the similar geometry of the P_5_^+^-core in all cations, the respective ^1^ *J*(PP) and ^2^ *J*(PP) coupling constants deviate only marginally. However, the chemical shifts are strongly dependent on the substituent R attached to the RP_5_Cl^+^-cage (Fig. 8). The P_A_ and P_B_ atoms exhibit characteristic low field resonances at approximately –275 ppm. The assignment of the A and B part to the respective P nuclei is based on the observed coupling pattern. First, the non-symmetrically substituted P_5_^+^-cage is divided by a plane spanned by the tetra-coordinated and both adjacent P atoms into a H_Cl_- and H_R_-hemisphere (Fig. 9).

The H_Cl_-hemisphere contains the chloro-substituent and the H_R_-hemisphere the alkyl- or aryl-substituent. Within the series of cations **32a–h** ^+^ the P atom located in the H_Cl_-hemisphere shows values of ^1^ *J*(PP) and ^2^ *J*(PP) coupling constants which are reminiscent of those of P_5_X_2_^+^-cages **26a–c** ^+^.^36,37^ Accordingly, the P atom located in the H_R_-hemisphere reveals one- and two-bond P–P coupling constants similar to the values observed for the respective R_2_P_5_^+^-cages. In addition, the former group of P atoms experiences the spatial proximity of the chloro-group, and, therefore, shows similar chemical shifts (marked in green, Fig. 8). In contrast, the P atoms in the H_R_-hemisphere show resonances in a much larger chemical shift range. This is attributed to the distinct electronic parameters of the substituents. They affect the chemical shifts of the P atoms most likely through “cross-ring through space” interactions of the lone pairs on P atoms and the respective group R.^45^ For RP_5_Cl^+^-cages featuring alkyl-substituents R (**32a–e** ^+^) the resonances of the P atoms adjacent to the phosphonium moiety (marked in blue, Fig. 8) are shifted stepwise to lower field with a decreasing positive inductive effect of the substituent (from 44 ppm (**32a** ^+^) to 81 ppm (**32e** ^+^)). This is in agreement with the increased shielding of a P atom caused by additional alkyl-moieties in the γ-position relative to the P nuclei. This trend was previously termed γ-effect.^46^ In contrast, the chemical shifts of tetra-coordinated P atoms (marked in red, Fig. 8) exhibit an almost inverse trend (from 99 ppm (**32a** ^+^) to 69 ppm (**32e** ^+^)). This high-field shift correlates with an increasing number of hydrogen atoms at the α-carbon atom of the substituent. This constitutes a characteristic feature of phosphonium moieties and was termed *a*-effect.^47^ Overall, these influences are reflected in a change of the spin system between **32e–h** ^+^ featuring aryl- and methyl-substituents (ABMX_2_ spin system) and those bearing alkyl-substituents **32a–d** ^+^ (ABM_2_X spin system).

**Scheme 9:**
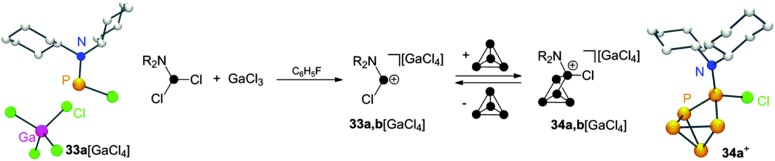
Reaction of R_2_NPCl_2_ (R = Cy, i-Pr) with GaCl_3_ and P_4_ and equilibrium of **34a,b** ^+^ with **33a,b** ^+^ and P_4_ (middle) and molecular structure of **33a**[GaCl_4_] (left) and **34a** ^+^ (right).

Employing dichlorophosphanes R_2_NPCl_2_ (R = Cy, i-Pr) in combination with GaCl_3_ in reactions with P_4_ gave distinct results. In mixtures of R_2_NPCl_2_ (R = Cy, i-Pr) and GaCl_3_ the corresponding phosphenium ions **33a,b** ^+^ are the only observable species.^20*a*^ Indicative of their formation is a resonance in the ^31^P NMR which is shifted to remarkable low field.^18^ It is highly influenced by the nature of the respective anion (compare **33a**[GaCl_4_]: *δ* = 310 ppm, **33a**[Ga_2_Cl_7_]: *δ* = 350 ppm). The GaCl_4_^–^ salt of **33a** ^+^ can be isolated and constitutes a rare example of a structurally characterized mono-amino substituted phosphenium ion (Scheme 9). Upon reacting phosphenium ions **33a,b** ^+^ with P_4_ insertion into a P–P bond is observed giving the *C*_S_-symmetric RP_5_Cl^+^-cage cations **34a,b** ^+^. However, these cages are in equilibrium with the respective free phosphenium ions and P_4_ which hampers the isolation of pure compounds **34a,b**[GaCl_4_]. The observation of an equilibrium can be attributed to the relative stability of free **33a,b** ^+^. A similar reversibility of the phosphenium ion insertion was observed in the case of RP_5_Cl^+^ compounds. The addition of coordinating solvents like acetonitrile to solutions of **32**[ECl_4_] (E = Ga, Al) decomposes the respective metallate anion *via* chloride liberation. Nucleophilic attack of free chloride anions on **32** ^+^ yields mainly the starting materials P_4_ and RPCl_2_ (R = alkyl, aryl) in a back reaction.

It is interesting to note that a reaction between the two-fold amino-substituted phosphenium ion [(i-Pr_2_N)_2_P]^+^ (**35** ^+^) and P_4_ was not observed.^20*a*^ This is attributed to a significantly lowered electrophilicity of **35** ^+^ compared to **33a,b** ^+^.^19^ Also, diamino-phosphenium ions of type **35** ^+^ reveal frontier orbitals comparable to those of allyl-anions^48^ with the HOMO mainly located at the N atoms, and, thus, are not ambiphilic at the P moiety.

**Scheme 10:**
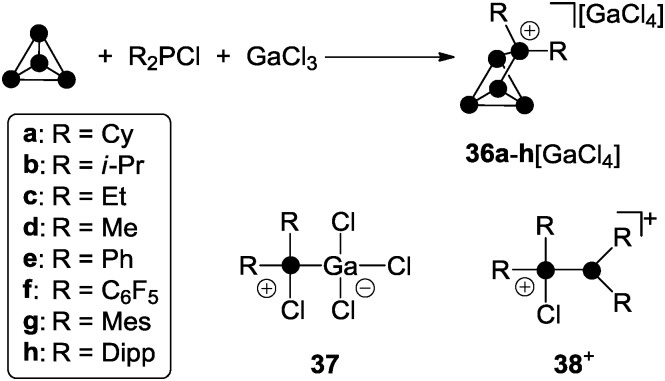
Preparation of compounds **36a–h**[GaCl_4_] from P_4_, R_2_PCl and GaCl_3_ in fluorobenzene (R = aryl) or according to a solvent free approach (R = alkyl) and species **37** and **38** ^+^ commonly observed in mixtures of R_2_PCl and GaCl_3_.

R_2_P_5_[GaCl_4_] cage compounds **36**[GaCl_4_] featuring two alkyl- or aryl-substituents R are obtained in high yield *via* the reaction of chlorophosphanes R_2_PCl, GaCl_3_ and P_4_ (Scheme 10).^49^

The Lewis acid–base adduct **37** and phosphanylphosphonium ion **38** ^+^ are commonly formed in mixtures of chlorophosphanes and GaCl_3_ in various stoichiometries.^22*b*^ Both convert into each other *via* equilibria involving free R_2_PCl and GaCl_3_.^49,22b^ Cations of type **38** ^+^ serve as phosphenium ion sources in the presence of P_4_ allowing for the formation of R_2_P_5_^+^-cage cations **36** ^+^. Most likely, this proceeds in analogy to quantum chemical calculations on the mechanism of the formation of MeP_5_Cl^+^ cage **32e** ^+^.^39^ In contrast to dichlorophosphanes, however, the reaction conditions for the formation of R_2_P_5_^+^-cages **36a–h** ^+^ depend strongly on the substituent R. In the case of chlorophosphanes featuring aryl substituents R, the reactions proceeds smoothly at ambient temperature in fluorobenzene solution. A significant decrease in reaction time is observed with increasing steric bulk of the substituents (Dipp > Mes > C_6_F_5_ > Ph). For the preparation of R_2_P_5_^+^-cages **36a–d** ^+^ featuring alkyl-substituents R, solvent-free conditions are necessary. Mixtures of R_2_PCl (R = Cy, i-Pr, Et, Me) and GaCl_3_ in a 1 : 1 stoichiometry form melts at ambient temperature.^28^ Upon addition of P_4_ to these melts, the formation of the corresponding cage compounds **36a–d**[GaCl_4_] is observed. With increasing steric demand of the substituents R (Cy > i-Pr > Et > Me) extended reaction times and higher temperatures (100 °C to 150 °C) are required. The different reactivity of alkyl- and aryl-substituted phosphanes in the synthesis of R_2_P_5_-cage compounds of type **36**[GaCl_4_] can be rationalized in terms of the different Lewis acidities of the corresponding phosphenium ions. The Lewis acidity is reflected *e.g.* by their distinct fluoride ion affinities (*e.g.* Me_2_P^+^: FIA = 959 kJ mol^–1^, Ph_2_P^+^: FIA = 838 kJ mol^–1^).^19,50^ This necessitates a more Lewis acidic environment for the transfer of a phosphenium ion featuring alkyl-groups, which is realized in a solvent free medium.

**Fig. 10:**
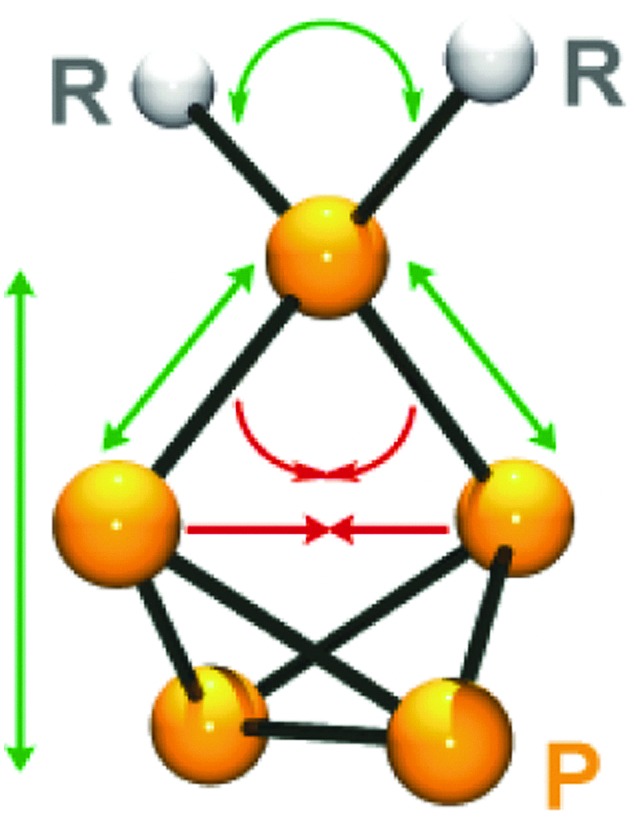
Influence of the steric demand of substituents R on the structural parameters of the P_5_^+^-cage core in cations of type **36** ^+^; upon increasing the steric demand of R green arrows indicate increasing angles and distances and red arrows indicate declining angles and distances.

The molecular structures of all compounds of the series **36a–h**[GaCl_4_] were determined by single crystal X-ray structure determination. This allowed for an in-depth evaluation of the influence of substituents of distinct steric demand on the structural parameters of the P_5_-cage in the solid state (Fig. 10).

The phosphonium P atoms of cations of type **36** ^+^ show a distorted tetrahedral environment. If the steric demand of the substituent R is increased a stepwise increase in the corresponding C–P–C angle is observed from the sterically very bulky substituted Dipp_2_P_5_^+^ (**36h** ^+^) to the methyl-substituted derivative **36e** ^+^. This is accompanied by a decreasing P–P–P angle at the phosphonium moiety and stepwise increase in P–P bond lengths involving the phosphonium P atom.

As a consequence, the tetraphosphabicyclo[1.1.0]butane moieties display a more pronounced folding (distance between both P atoms adjacent to the phosphonium P atom decreases) and the P_5_^+^-cages are stretched (distance between the bridgehead P–P bond and the phosphonium P atom increases).

**Fig. 11:**
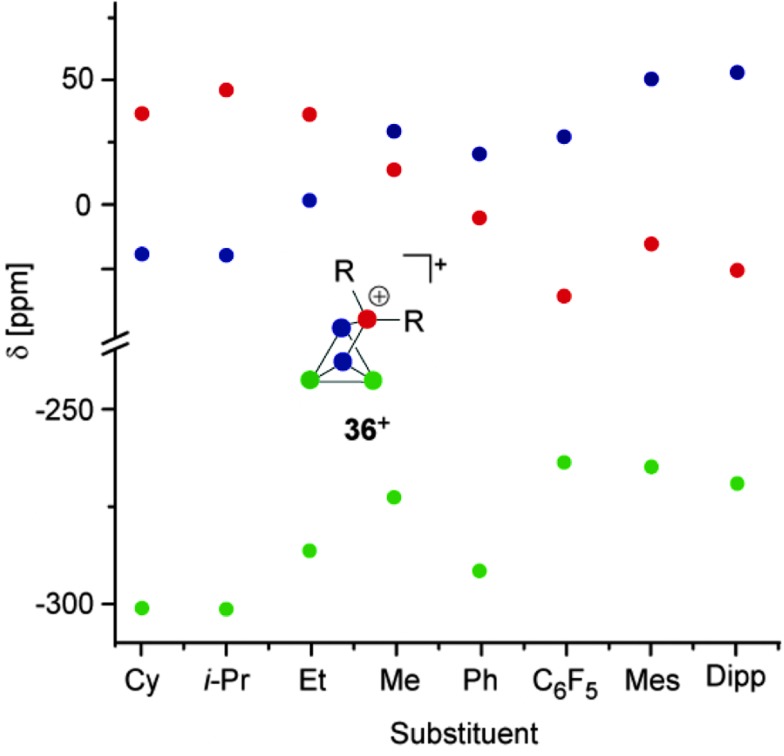
Plot of the ^31^P NMR chemical shifts of R_2_P_5_^+^-cages **36a–h** ^+^ *versus* their alkyl- or aryl-substituent.

**Scheme 11:**
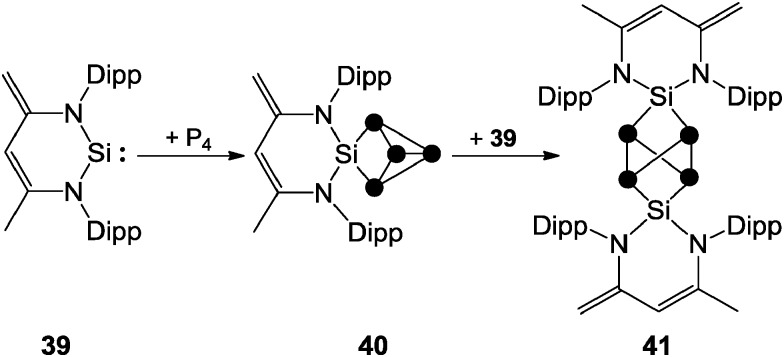
Stepwise insertion of zwitterionic silylene **39** into P–P bonds of P_4_ yielding SiP_4_-cage compound **40** and Si_2_P_4_-cage compound **41**.

The ^31^P NMR spectra of cage cations **36** ^+^ show A_2_M_2_X or A_2_MX_2_ spin systems in accordance with their *C*_2V_ symmetry and are comparable to those observed for the P_5_X_2_^+^ cages **26a–c** ^+^ (Fig. 11). The observation of two different spin systems for R_2_P_5_^+^-cages of type **36** ^+^ may be explained in terms of different steric and electronic influences of the alkyl- or aryl-substituent R. In the series of alkyl-substituted R_2_P_5_^+^-cages (**36a** ^+^ to **36d** ^+^) the resonances of the phosphonium P atoms are shifted to higher field and the resonances of the adjacent P atoms are shifted to lower field. This can be explained in terms of a combination of *a*-effect and γ-effect (*vide infra*).^46,47^ The resonances of the tetra-coordinated P atoms in aryl-substituted cations **36e–h** ^+^ are shifted to higher field compared to those of the corresponding P atoms in cages **36a–d** ^+^. This is due to a positive mesomeric effect, namely the donation of π-electron density from the aryl substituents to the lobes of the anti-bonding σ*(P–P) orbitals at the phosphonium moiety.^47*a*^ Some main group centered, predominantly electrophilic ambiphiles react with P_4_ *via* multiple insertions into P–P bonds of the P_4_ tetrahedron. This is exemplified by SiP_4_-cage compound **40**, which is obtained by the reaction of P_4_ with zwitterionic silylene **39**. This compound reacts with a second equivalent of **39** to give the Si_2_P_4_-cage compound **41** (Scheme 11).^14^ The second insertion takes place at a P–P bond opposing the initially inserted main group element. The related product **4** was obtained by the reaction of P_4_ with a low valent Al(i) species (Fig. 2).^13^

**Scheme 12:**
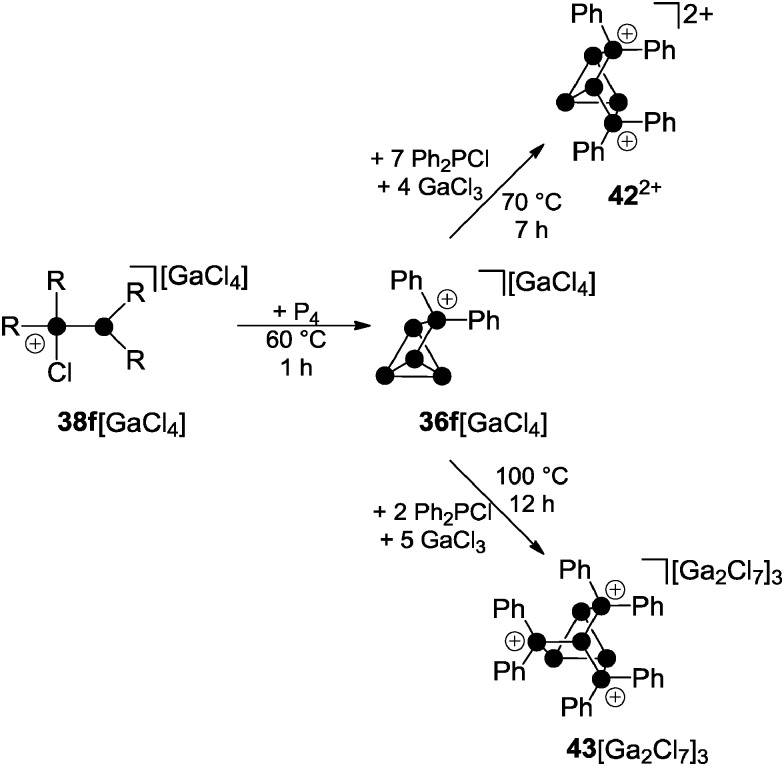
Stepwise insertion of Ph_2_P^+^-phosphenium ions into P–P bonds of P_4_ yielding Ph_4_P_6_^2+^-cage cation **42** ^2+^ and Ph_3_P_7_^3+^-cage compound **43**[Ga_2_Cl_7_]_3_.

Distinct results were obtained in the investigation of multiple insertions of phosphenium ions into P–P bonds of P_4_. In this context, solvent-free mixtures of P_4_, Ph_2_PCl and GaCl_3_ in various stoichiometries and at different temperatures were investigated. A 1 : 1 : 1 mixture yields quantitatively the Ph_2_P_5_^+^-cage compound **36f**[GaCl_4_] after 45 min at 60 °C (Scheme 12).^51^

The Ph_4_P_6_^2+^-cage cation **42** ^2+^ is observed in a mixture of 1 : 8 : 5 stoichiometry (P_4_ : Ph_2_PCl : GaCl_3_) as the main product after a reaction time of seven hours at 70 °C. The ^31^P NMR spectrum of **42** ^2+^ shows a characteristic ABMM′XX′ spin system which is in accordance with the insertion of a second Ph_2_P^+^-phosphenium ion into a P–P bond adjacent to the phosphonium moiety in **36f** ^+^. Two second-order resonances corresponding to an AA′XX′X′′X′′′ spin system are expected for the isomer of **42** ^2+^ formed *via* formal insertion into two opposing P–P bonds of P_4_. Such a species is not formed in the melt reaction. The formation of dication **42** ^2+^ can only be observed if the ratio of Ph_2_PCl and GaCl_3_ is higher than 0.5. In these mixtures, the dominant gallium species is GaCl_4_^–^; hence, the melt can be considered as basic medium. In a more Lewis acidic melt, composed of P_4_, Ph_2_PCl and GaCl_3_ in a 1 : 3 : 6 stoichiometry, the tricationic Ph_6_P_7_^3+^-cage **43** ^3+^ is formed exclusively. Large single crystals of **43** ^3+^ as a heptachlorodigallate salt are formed in the respective melt after 12 h at 100 °C. Cation **43** ^3+^ features a nortricyclane-type (tricyclo[2.2.1.0^2.6^]heptane) framework. It is composed of a basal ring of three-coordinated P atoms, three tetra-coordinated P atoms at the bridging positions and a three-coordinated P atom at the apex of the cage. This skeleton is reminiscent of the trianionic phosphide P_7_^3–^,^52^ several polyphosphanes R_3_P_7_^53^ and many polyphosphorus-chalcogenides like *e.g.* P_4_S_3_.^54^ The ^31^P NMR spectrum of **43** ^3+^ shows an AA′A′′BXX′X′′ spin system resulting from the *C*_3_ symmetry of the cage. A ^2^ *J* or ^3^ *J* P–P bond coupling to the apex of the cage is not observed which might be a result of the adjacent phosphonium P atoms. This leads to a first-order quartet resonance for the apical P atom. The highly electrophilic cation **43** ^3+^ is stable only in the presence of excess GaCl_3_. This prevents the detrimental presence of chloride anions which decompose **43** ^3+^ by nucleophilic attack and subsequent degradation *via* **42** ^2+^ to **36f** ^+^. This illustrates that the consecutive insertion of up to three Ph_2_P^+^-moieties into P–P bonds of P_4_ is directed by the Lewis acidity of the reaction mixture.

## 7. Cationic polyphosphorus cages featuring four-membered heterocycles

**Scheme 13:**
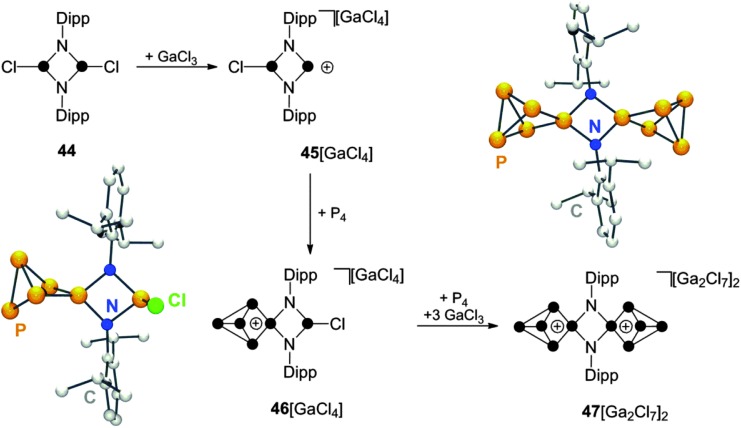
Stepwise synthesis of N_2_P_10_-cage compound **47**[Ga_2_Cl_7_]_2_ *via* insertion of phosphenium ions generated *in situ* by the reaction of diphosphadiazane **44** with GaCl_3_.

Cyclic diaminohalophosphanes are important precursors for the preparation of cyclic phosphenium ions *via* halide abstraction.^55^ Within this class of compounds, phosphazanes, like the diphosphadiazane **44**, are of particular interest (Scheme 13). These compounds feature two chloro-substituted P moieties and, thus, offer a versatile reactivity.^56^ The diphosphadiazenium ion **45** ^+^ is generated from **44** upon chloride abstraction with GaCl_3_. Solutions of **45** ^+^ are characterized by a bright red colour and the ^31^P NMR spectrum shows a broad resonance at characteristic low field (*δ* = 242.3 ppm) indicating the formation of a di-coordinated P moiety. Subsequent addition of P_4_ to this solution leads to discolouration and quantitative formation of the P_5_^+^-cage compound **46**[GaCl_4_].^57^ The molecular structure of cation **46** ^+^ shows a planar four-membered (NP)_2_ ring and an almost orthogonal oriented P–Cl bond (Scheme 13). This arrangement is also reflected by the A_2_MVXZ spin system observed in the ^31^P NMR spectrum of *C*_S_-symmetric cation **46** ^+^. Interestingly, the P_5_^+^-cage does not couple with the chloro-substituted P atom resulting in the observation of a singlet resonance for the latter. This P–Cl functionality was used for the *in situ* generation of a phosphenium ion upon addition of three equivalents of GaCl_3_ to the reaction mixture. The resulting dicationic intermediate was not detected. However, upon addition of P_4_, the formation of the corresponding insertion product **47** ^2+^ is observed. The ^31^P NMR spectrum of **47** ^2+^ shows an A_2_MX_2_ spin system which is consistent with two *C*_2V_-symmtric P_5_^+^-cages bridged by two imido-groups. The dication can be isolated as heptachlorodigallate salt **47**[Ga_2_Cl_7_]_2_ and the molecular structure of the N_2_P_10_-cage was confirmed by single crystal structure determination (Scheme 13). This illustrates that the stepwise insertion of the disguised bifunctional Lewis acid [DippNP]_2_^2+^ into P–P bonds of two P_4_ tetrahedra can be mediated by the Lewis acidity of the reaction mixture. The utilization of an excess of GaCl_3_ allows for the preparation of the more electrophilic, higher charged species **47** ^2+^, similar to the reaction sequence yielding **43** ^3+^ (Scheme 12).

It is interesting to note that related NHC analogues, five-membered 1,3,2-diazaphospholenium ions, do not react with P_4_ under various reaction conditions^58^ similar to acyclic, diamino-phosphenium ion (i-Pr_2_N)_2_P^+^ (*vide infra*). This indicates that the strained four-membered ring geometry present in diphosphadiazenium ions is crucial for its reactivity towards P_4_.

**Scheme 14:**
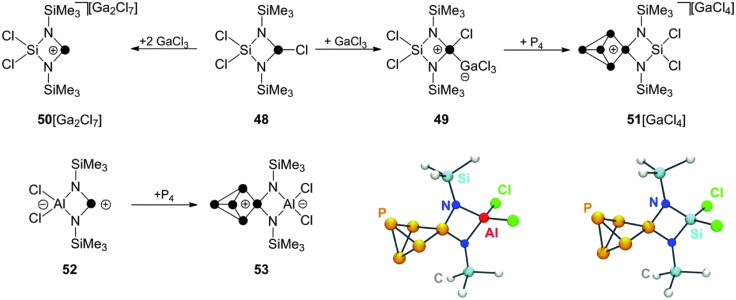
Preparation of P_5_^+^-cage cation **51** ^+^ from P_4_, GaCl_3_ and chlorophosphane **48** (top) and preparation of zwitterionic P_5_-cage compound **53** from P_4_ and zwitterionic phosphenium ion **52**.

Other cyclic, four-membered phosphorus containing heterocycles can be employed in reactions with P_4_ as well.^59^ The cyclic chlorophosphane **48**, featuring a SiCl_2_-backbone,^60^ reacts with GaCl_3_ to give the corresponding Lewis acid–base adduct **49** (Scheme 14). The formation of related phosphenium ion **50** ^+^ is observed only upon addition of a second equivalent of GaCl_3_. This can be explained by the suppression of detrimental concentrations of nucleophilic chloride anions through the formation of Ga_2_Cl_7_^–^.

Cation **50** ^+^ is not stable in solution and decomposes *via* Lewis acid mediated Me_3_SiCl elimination. However, the insertion reaction with P_4_ requires only the use of one equivalent of GaCl_3_. In 1 : 1 : 1 mixtures of **48**, GaCl_3_ and P_4_ the corresponding P_5_^+^-cage compound **51**[GaCl_4_] is formed slowly within four days presumably due to the presence of small amounts of **50** ^+^ formed from **49** in a series of equilibrium reactions.^59^ The related zwitterionic phosphenium ion **52** features a formally anionic AlCl_2_-backbone.^60^ It reacts with P_4_ in toluene giving the formally neutral P_5_-cage compound **53**. A conversion of only 30% to the respective product is observed in the reaction mixture, presumably due to the low electrophilicity of **52**. However, the developed synthetic protocol includes removal of unreacted starting materials **52** and P_4_ by sublimation which can be used in additional synthetic cycles increasing the overall isolated yield.

## 8. Cationic polyphosphorus-chalcogen cages

**Scheme 15:**
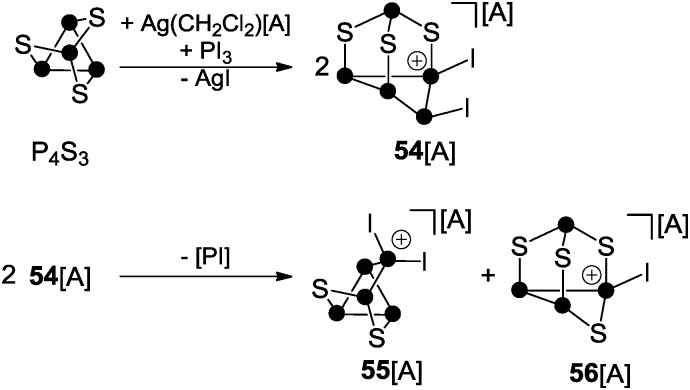
Reaction of P_4_S_3_ with *in situ* generated phosphenium ion PI_2_^+^; A = Al(OC(CF_3_)_3_)_4_.

A multitude of phosphorus-chalcogenides have been characterized to date and many of their structural motifs are displayed even in undergraduate textbooks.^61^ However, until recently, only very few examples of polyphosphorus-chalcogen cations were known which was due to the lack of established synthetic routes for their preparation. To the best of our knowledge only three distinct protocols have been reported so far. The first is based on the reaction of P_4_S_3_ with *in situ* generated phosphenium ion PI_2_^+^ (Scheme 15).^44*b*^

The phosphenium ion formally inserts into a P–P bond of the basal P_3_-ring accompanied by migration of one of the iodo-substituents to an adjacent P atom giving cation **54** ^+^. However, **54** ^+^ is not stable and subsequently disproportionates *via* an unknown reaction pathway to form **55** ^+^ and **56** ^+^. This process involves the extrusion of a very reactive iodo-phosphinidene [PI] and redistribution of the sulfur atoms.

**Scheme 16:**
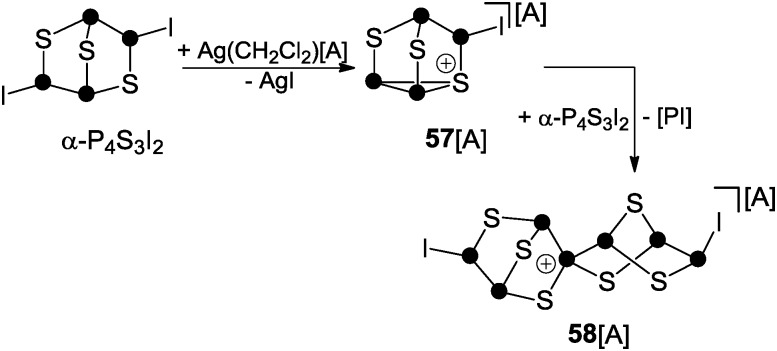
Reaction of α-P_4_S_3_I_2_ with Ag(CH_2_Cl_2_)[A], A = Al(OC(CF_3_)_3_)_4_.

The second protocol is based on halide abstraction from α-P_4_S_3_I_2_ with Ag(CH_2_Cl_2_)[Al(OC(CF_3_)_3_)_4_] and yields the spiro-cyclic cage cation **58** ^+^ (Scheme 16).^62^ The initial step involves the formation of **57** ^+^ *via* iodide abstraction from α-P_4_S_3_I_2_. Cation **57** ^+^ subsequently reacts with a second equivalent of α-P_4_S_3_I_2_ and this in association with the formal extrusion of phosphinidene [PI] gives rise to spiro-cyclic cage **58** ^+^. However, detailed information on the mechanism of the formation of **58** ^+^ was not gained. The structural motif of this cation is unprecedented and contains the first tetra-coordinated P atom exclusively bonded to P and S atoms.^62^

**Scheme 17:**
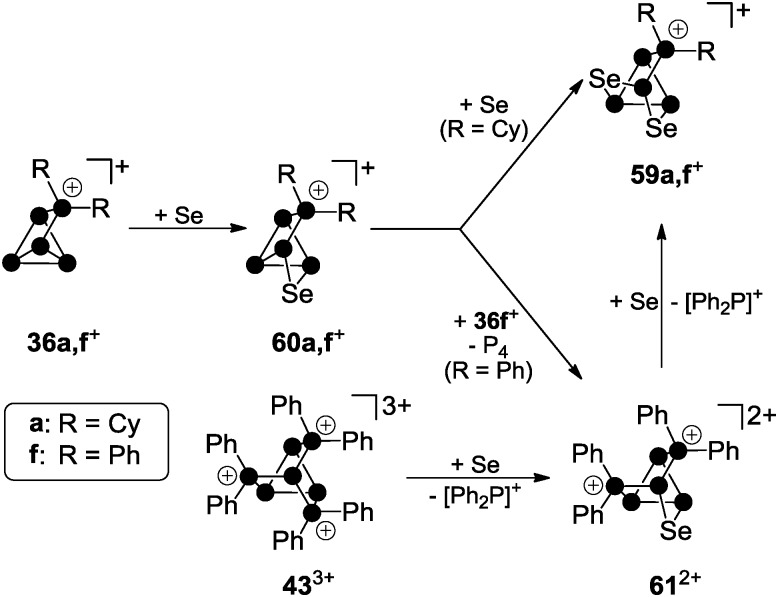
Stepwise insertion of selenium atoms into P–P bonds of **36a,f** ^+^ and stepwise substitution of [Ph_2_P]^+^-moieties in **43** ^3+^ by selenium atoms giving the nortricyclane-type polyphosphorus-chalcogen cage cations **59a,f** ^+^ and **61** ^2+^.

Recently, a third approach garnered interest which is based on using cationic polyphosphorus cages as starting materials for the preparation of cationic polyphosphorus-chalcogen cages. They constitute potentially versatile reagents due to the multitude of distinctly substituted derivatives which are all conveniently obtained in one step procedures from white phosphorus.^49^ Chalcogenation reactions of R_2_P_5_^+^-cage compounds **36a**[GaCl_4_] and **36f**[GaCl_4_] with elemental grey selenium yield the corresponding polyphosphorus-selenium cages **59a**[GaCl_4_] and **59f**[GaCl_4_] (Scheme 17). Both are obtained at elevated temperatures (110–150 °C) following a solvent-free protocol. In some cases, the addition of one equivalent of GaCl_3_ is beneficial since it lowers the melting point of the respective melt. Both cations are formed upon insertion of two selenium atoms into two P–P bonds adjacent to the phosphonium moieties in **36a,f** ^+^.

**Fig. 12:**
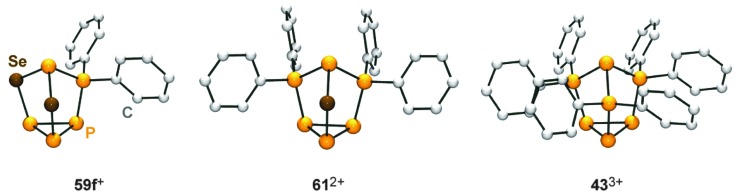
Nortricyclane type molecular structures of the related, polyphosphorus cations **59f** ^+^, **61** ^2+^ and **43** ^3+^.

Their structural motif resembles that of nortricyclane, with a basal P_3_-ring, the tetra-coordinated P atom and the selenium atoms occupying the bridging positions, and one P atom defining the apex of the cage. This class of compounds feature interesting ^31^P and ^77^Se NMR characteristics. Cages **59a,f** ^+^ reveal an AM_2_OX spin system for the *C*_S_-symmetric isotopomer without a ^77^Se nucleus. These resonances are superimposed by the *C*_1_-symmetric isotopomer featuring one ^77^Se atom in one of the bridging positions. This isotopomer gives rise to an AMNOXZ spin system which is highly influenced by higher order effects. However, in the case of **59a** ^+^, the spin systems of both isotopomers were successfully simulated allowing for the exact determination of chemical shifts and coupling constants. A series of experiments employing varying temperatures, reaction times and stoichiometries gave meaningful insights into the mechanism of the chalcogenation. These experiments indicate that the insertion of Se atoms into P–P bonds of **36a,f** ^+^ proceeds in a stepwise manner *via* the intermediates **60a,f** ^+^. In the case of alkyl-substituted cage **36a** ^+^ the insertion of a second equivalent grey selenium is fast, yielding the respective product **59a** ^+^ quantitatively. If the aryl-substituted starting material **36f** ^+^ is employed, the intermediate formation of dication **61** ^2+^ is observed. This species forms *via* the transfer of a [Ph_2_P]^+^ moiety from a second equivalent of **36f** ^+^ to the reactive intermediate **60f** ^+^. Due to the higher stability of the corresponding phosphenium ion Ph_2_P^+^,^19,50^ this transfer is faster than the insertion of the second selenium atom. Subsequently, one of the [Ph_2_P]^+^-moieties of **61** ^2+^ is substituted by a selenium atom giving rise to **59f** ^+^. The formally liberated Ph_2_P^+^-phosphenium ion is not stable and reacts with a GaCl_4_^–^ anion to give the Lewis acid–base adduct **37** (Ph_2_PCl–GaCl_3_). This is in accordance with the observation of only 50% conversion and the quantitative formation of P_4_ and **37** or the respective oxidation product Ph_2_P(Se)Cl–GaCl_3_ in the case of reactions involving **36f** ^+^ as a starting material. The targeted preparation of **61** ^2+^ as GaCl_4_^–^ salt is achieved by utilizing a 2 : 1 stoichiometry of **36f** ^+^ and grey selenium. Another synthetic approach for the preparation of **61** ^2+^ is the targeted substitution of one [Ph_2_P]^+^-moiety in the tricationic cage **43** ^3+^. This is achieved by reacting **43** ^3+^ with grey selenium under solvent-free conditions (Scheme 17).^49^ Dication **61** ^2+^ was comprehensively characterized by X-ray crystallography (Fig. 12) as well as ^31^P and ^77^Se NMR spectroscopy. The ^31^P NMR spectrum reveals a characteristic AA′MOXX′-spin system for the isotopomer without a ^77^Se nucleus which is superimposed by the respective AA′MOXX′Z-spin system of the ^77^Se containing species.

**Fig. 13:**
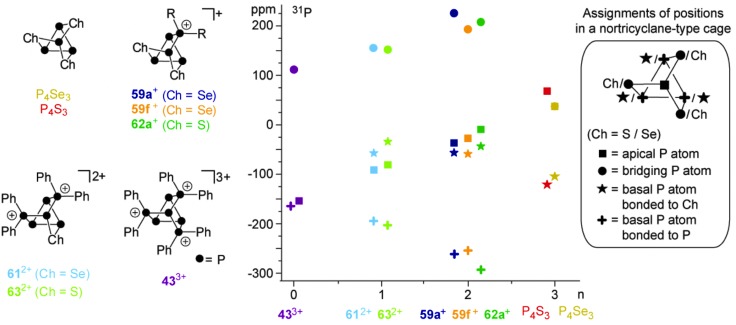
Family of cationic polyphosphorus-chalcogen cages formally derived from stepwise isolobal exchange of [Ch] by [R_2_P]^+^ units in P_4_Ch_3_ (Ch = Se, S, left), their ^31^P NMR shifts *versus* the number of chalcogen atoms (*n*, middle) and the assignment of P or Ch atoms to the positions of a nortricyclane-type cage (right).

A similar reactivity was observed for reactions of the P_5_^+^-cage **36a** ^+^ or the P_7_^3+^-cage **43** ^3+^ with elemental α-S_8_.^49^ The polyphosphorus cation **43** ^3+^ and cationic polyphosphorus-chalcogen cages **61** ^2+^ and **59f** ^+^ are formally derived from the stepwise isolobal exchange of [Se] atoms by [Ph_2_P]^+^ units in the bridging positions of the nortricyclane-type structure of P_4_Se_3_. This allows for an in-depth study of the ^31^P NMR characteristics of the whole series of compounds and a correlation with the observed structural features in the solid state. Fig. 13 shows the dependence of the chemical shifts of **43** ^3+^, **61** ^2+^ and **59f** ^+^, the related sulfur-containing cages **62a** ^+^ and **63** ^2+^, and P_4_Ch_3_ (Ch = Se, S)^63^ on the number of chalcogen atoms in the corresponding molecules. The stepwise exchange of tetra-coordinated P atoms in **43** ^3+^ by Se or S atoms is accompanied by a high-field shift of the resonances of the P atoms of the basal three-membered ring. The chemical shifts of basal P atoms in nortricyclane-type cages are influenced by the exocyclic angles of the P_3_-ring.^63^ The observed high-field shift correlates well with decreasing exocyclic angles observed in the solid state structures of the respective compounds. The resonances of apical P atoms exhibit the widest range of chemical shifts and reveal a stepwise down-field shift upon the substitution of tetra-coordinated P atoms by chalcogen atoms. This is consistent with different electronegativities of directly bonded phosphorus or chalcogen atoms. Moreover, apical P atoms show a high dependency of their chemical shift on elongation or compression of the nortricyclane framework.^64^ Elongation is accompanied by a decrease in the P–P–P angles involving the apical P atom. This increases the s-orbital contribution to the lone pair of electrons and leads to an upfield shift of the corresponding resonance in the ^31^P NMR spectrum.^65^ On this basis, the observed downfield shift indicates a stepwise elongation of the cages which is observed in the respective molecular structures in the solid state.

## 9. Nucleophilic fragmentation of cationic polyphosphorus cages

**Scheme 18:**
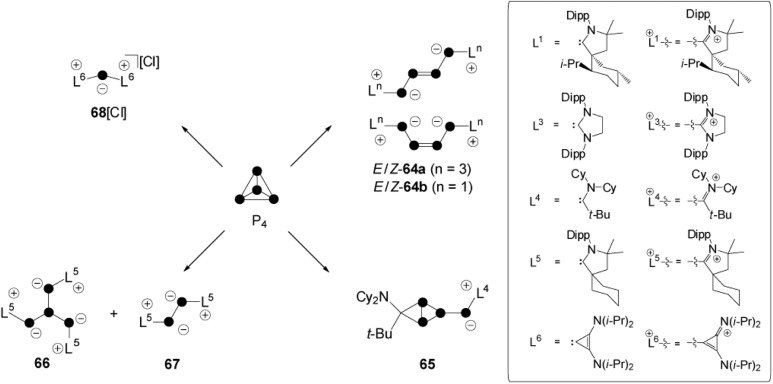
Carbene-induced transformation and fragmentation reactions of P_4_.

The activation of white phosphorus with carbenes, which belong to the class of predominantly nucleophilic ambiphiles, displays one of the most diverse fields of P_4_ chemistry.^6^ The *cyclo*-triphosphirene derivative **C** constitutes a key intermediate in all transformations, independent of the characteristic of the respective carbene employed (Fig. 1). However, intermediate **C** is elusive and distinct reaction pathways occur depending on the electronic and steric features of carbene **L** (Scheme 18). Bertrand and co-workers reacted P_4_ with carbenes **L^1^** and **L^3^** in a 1 : 2 stoichiometry and obtained *E*/*Z* isomers **64a,b** *via* an intermediate of type **C**.^12^ Bicyclic species **65** is the result of a cyclo-addition reaction involving the phosphorus double bond of an intermediate of type **C** and the alkyl amino carbene **L^4^**.^66^ Compound **66** results from a ring-opening reaction of an intermediate **C** with two equivalents of **L^5^**.^66^ This reaction is accompanied by the formation of **67** as a side product. This P_2_-species is formed by the formal [2+2] fragmentation of P_4_ by carbene **L^5^**. A [3+1]-fragmentation of the P_4_ tetrahedron was achieved using the sterically less demanding carbene **L^6^** in a reaction with P_4_ in a 3 : 1 stoichiometry.^66^ The P_1_-fragment was identified as **68** ^+^ and isolated as chloride salt. The presence of chloride anions is explained by the decomposition of CHCl_3_ solvent molecules.

A compound of unknown constitution featuring a P_3_ moiety was indicated in the ^31^P NMR spectrum of the respective reaction mixture.^66^

**Scheme 19:**
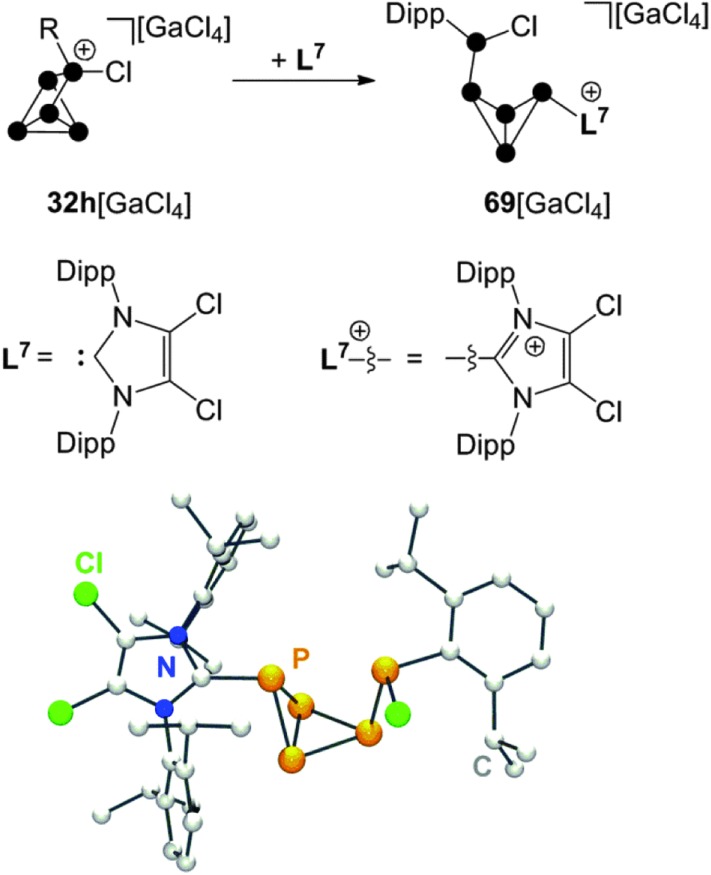
Reaction of **32h**[GaCl_4_] with carbene **L^7^** in a 1 : 1 stoichiometry (top) and molecular structure of cation **69** ^+^ (bottom).

A combination of phosphenium ion and carbene mediated P_4_ activation constitutes a novel, potentially versatile approach for the preparation of cationic polyphosphorus cages. This strategy allows for the preparation of polyphosphorus cations featuring imidazoliumyl-substituents. These substituents are valuable for two purposes. First, they serve well for the stabilization of cations by delocalization of the positive charge.^70^ Second, they stabilize low-coordinated P moieties by reducing the nucleophilicity of directly bonded P atoms.^71^ The reaction of P_5_^+^-cage compound **32h**[GaCl_4_] with carbene **L^7^** in a 1 : 1 stoichiometry yields the bicyclo[1.1.0]tetraphosphane **69**[GaCl_4_] (Scheme 19).^67^ The bicyclic framework is substituted with an imidazoliumyl-group in an *exo*-position and a phosphanyl-group in an *endo*-position. This is reminiscent of the intermediate **31** observed in the formation of RP_5_Cl^+^-cages. Cation **69** ^+^ features an ACEMX spin system indicating a non-symmetrical molecular structure due to hindered rotation around the P–P bond involving the Dipp-substituted P atom. The *endo*,*exo*-substitution of **69** ^+^ causes a short intermolecular distance between the Dipp- and the imidazoliumyl-substituted P atoms in the solid state (see molecular structure in Scheme 19). This spatial proximity is also indicated in solution by an extraordinarily large ^3^ *J*(PP) coupling constant of 244.6 Hz in the ^31^P NMR spectrum.

**Scheme 20:**
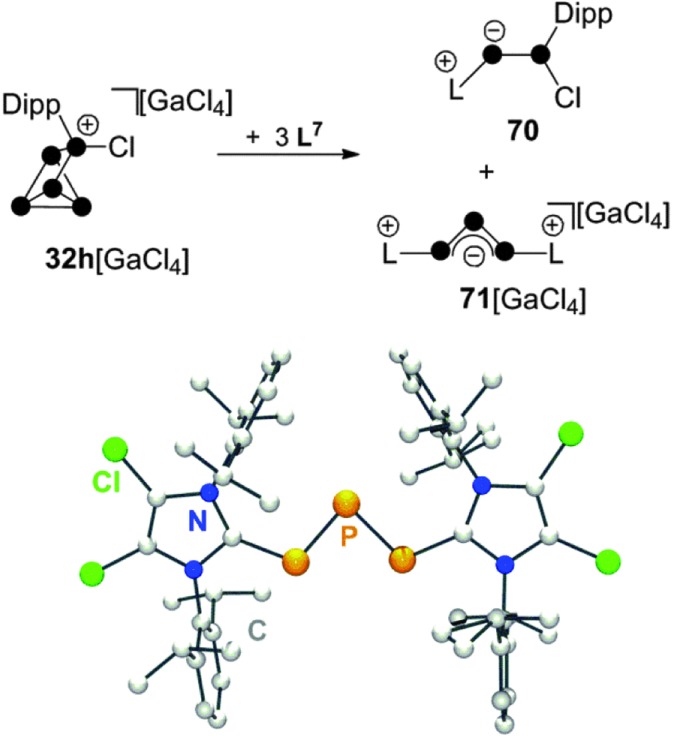
Reaction of **32h**[GaCl_4_] with carbene **L^7^** in a 1 : 3 stoichiometry (top) and molecular structure of cation **71** ^+^ (bottom).

The reaction of **32h**[GaCl_4_] with carbene **L^7^** in a 1 : 3 stoichiometry proceeds *via* a quantitative [3+2]-fragmentation of the P_5_^+^-cage (Scheme 20).^67^

**Scheme 21:**
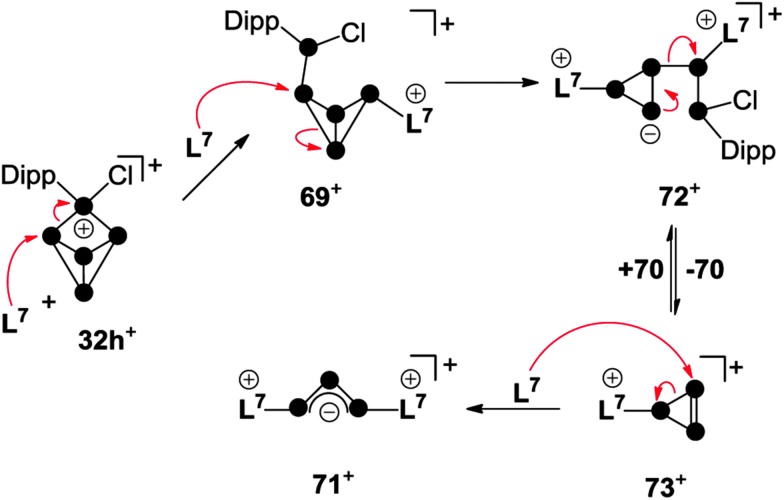
Reaction sequence for the carbene-induced [3+2]-fragmentation of P_5_^+^-cage **32h** ^+^.

The P_2_ fragment was identified as the neutral P_2_ species **70** featuring an inversely-polarized^68^ phosphaalkene moiety. The di-coordinated P atom bears a phosphanyl-substituent which originates from the tetra-coordinated P atom of starting material **32h** ^+^. The P_3_ fragment was identified as GaCl_4_^–^ salt of cation **71** ^+^ which features a chain of three di-coordinated P atoms terminated by two imidazoliumyl-substituents. This compound is characterized by a deep green colour that results from n → π* and π → π* transitions similar to those observed in diphosphenes.^69^ Quantum chemical calculations elucidated the bonding in **71** ^+^.^67^ The frontier orbital arrangement of the cation is closely related to the classical π-system of the C_3_-allyl anion. Thus, **71** ^+^ features a local triphosphaallylanion moiety substituted with imidazoliumyl-groups. The mechanism of the [3+2] fragmentation is explained by the reaction sequence in Scheme 21 on the basis of experimental evidence and quantum chemical calculations.^67^ The reaction of **32h** ^+^ with the first equivalent of **L^7^** yields the experimentally verified species **69** ^+^. The nucleophilic attack of **L^7^** occurs at a P atom adjacent to the phosphonium moiety in **32h** ^+^ and initiates a P–P bond cleavage. This reaction step is the reverse of the last step in the formation of RP_5_Cl^+^-cages (Fig. 7) and is in accordance with the observed reversibility of phosphenium ion insertion into P–P bonds of P_4_ (*vide infra*). The nucleophilic attack of a second carbene **L^7^** occurs at the *endo*-substituted P atom of **69** ^+^ and initiates a P–P bond cleavage in the respective P_3_-ring.

This yields intermediate **72** ^+^ according to quantum chemical calculations.^67^ Subsequently, this intermediate intramolecularly eliminates the P_2_ fragment **70**. This yields the elusive triphosphirene derivative **73** ^+^ which is related to the key intermediate **C** (Fig. 1) of carbene-induced P_4_ activation.^12,66^ The nucleophilic attack of a third carbene **L^7^** on the PP double bond of **73** ^+^ initiates a ring-opening and yields the second fragment **71** ^+^. The ease of fragmentation (high yields, multi-gram scale) together with the facile accessibility of cationic phosphorus cages from P_4_ and the multitude of carbenes available render this approach suitable for the preparation of a plethora of interesting polyphosphorus compounds.

## Abbreviations

aAAC: Acyclic alkyl amino carbene
Ab: Ambiphile
Ch: Chalcogen atom (Se or S)
El: Electrophile
Et: Ethyl
cAAC: Cyclic alkyl amino carbene
Cy: Cyclo-hexyl
Dipp: 2,6-Di-iso-propylphenyl
FIA: Fluoride ion affinity
HOMO: Highest occupied molecular orbital
i-Pr: Iso-propyl
LUMO: Lowest unoccupied molecular orbital
Me: Methyl
Mes*: 2,4,6-Tri-*tert*-butylphenyl
NHC: N-Heterocyclic carbene
Nu: Nucleophile
OTf: Triflate, trifluoromethylsulfonate
*t*-Bu: *tert*-Butyl
